# Diseases of the tongue: a review of etiologies and treatments

**DOI:** 10.1186/s40902-026-00511-1

**Published:** 2026-05-18

**Authors:** Young Heon Jeong, Heonwoo Lee, Kang-Min Ahn

**Affiliations:** 1 Department of Oral and Maxillofacial surgery, Asan medical Center, College of Medicine, University of Ulsan, Ulsan, Korea, Republic of; 2 Department of Pathology, Asan medical Center, College of Medicine, University of Ulsan, Ulsan, Korea, Republic of

**Keywords:** Tongue, Cancer, Precancerous lesions, Systemic disease, Congenital malformation, Benign tumor

## Abstract

**Objective:**

Tongue diseases are highly diverse and difficult to differentiate due to their varied etiologies. This clinical review summarizes various tongue diseases based on clinical experience and proposes appropriate treatment strategies, emphasizing the tongue’s multifunctional role and its impact on quality of life.

**Subjects and methods:**

This clinical review analyzes a broad spectrum of tongue diseases encountered in clinical practice. Lesions were categorized according to etiology, including infectious, inflammatory, benign and malignant neoplastic, precancerous, congenital, autoimmune, and systemic disease–related manifestations. Relevant literature was reviewed in conjunction with the authors’ clinical experience.

**Results:**

A total of 36 tongue diseases were classified, and the characteristic clinical features and treatment approaches for each condition were presented.

**Conclusion:**

In this clinical review, various tongue diseases are reviewed and treatments were suggested.

## Background

The tongue is the most versatile organ which is involved in speech, swallowing, chewing, expression, kissing and tasting. Unfortunately, many patients suffer from the diseases of the tongue and the complete remission of the diseases is sometimes not possible [1]. The diseases of tongue range from minor oral ulcer to cancer which is devastating if untreated. There are several articles have reviewed the diseases of the tongue [1, 2], however, there remains a need for a consolidated review that encompasses the wide variety of tongue conditions, as previous studies have often been limited in scope. This article aims to review a broad spectrum of tongue diseases based on the authors’ clinical experiences. A diverse range of pathological and symptomatic conditions is presented to aid in differential diagnosis.

## Methods

This study is presented as a narrative review combining the authors’ clinical experience with supporting literature. Clinically, the review is based on patients who presented with chief complaints of tongue discomfort and were referred to our tertiary general hospital between 2006 and 2025. For conditions encountered infrequently in clinical practice, the literature was supplemented by searches of PubMed using relevant search terms without date restriction. Articles were selected based on their clinical relevance and the authors’ judgment, consistent with the narrative review format. This approach was chosen to provide a practical, experience-based overview rather than a systematic or quantitative synthesis.

Grossly the diseases of the tongue could be divided into two parts. One is pathologic condition of the tongue organ itself, and the other is tongue pain and discomfort associated without any visible pathology. Usually, the second condition is related with systemic diseases or other autoimmune conditions. The pathologic condition includes vascular and lymphatic lesions, reactive and inflammatory conditions, infectious conditions, premalignant lesions, benign and malignant tumors and lesions related with systemic disease. In this review, the clinical sign and symptoms were reviewed and optimal treatments were suggested.

## Results

### Vascular and lymphatic lesions

#### Hemangioma (Fig. 1)

Hemangioma of tongue is relative common disease [3]. Tongue is rich in vessels and consisted with muscles. Because of the rich vascular component, hemangioma is commonly found. Hemangioma in early age could be regressed spontaneously [4]. However, some of the hemangioma in the tongue increases in size with age. Hemangiomas are benign vascular tumors characterized histopathologically by lobules of proliferating capillaries lined with plump endothelial cells during the proliferative phase, progressing to dilated vascular spaces lined by flattened endothelium and increased fibrosis in the involution phase (Fig. 2). Simple excision is enough for small, isolated lesions; however, huge sized hemangioma or less demarcated lesions are hard to remove because of risk of bleeding and functional deficit after glossectomy. In large sized hemangioma or venous malformation, local injection of sclerosing agents such as 3% sodium tetradecyl sulfate, ethanolamine oleate, pingyangmycin and bleomycin are indicated [5]. 3% Sodium tetradecyl sulfate (0.5–2 mL) is injected intralesionally to induce vascular thrombosis and subsequent fibrosis. Multiple sessions may be required depending on the lesion’s size and clinical response. The other treatments for large sized hemangioma are liquid nitrogen cryotherapy, low-temperature plasma radiofrequency and LASER therapy [6–8].

**Fig. 1:**
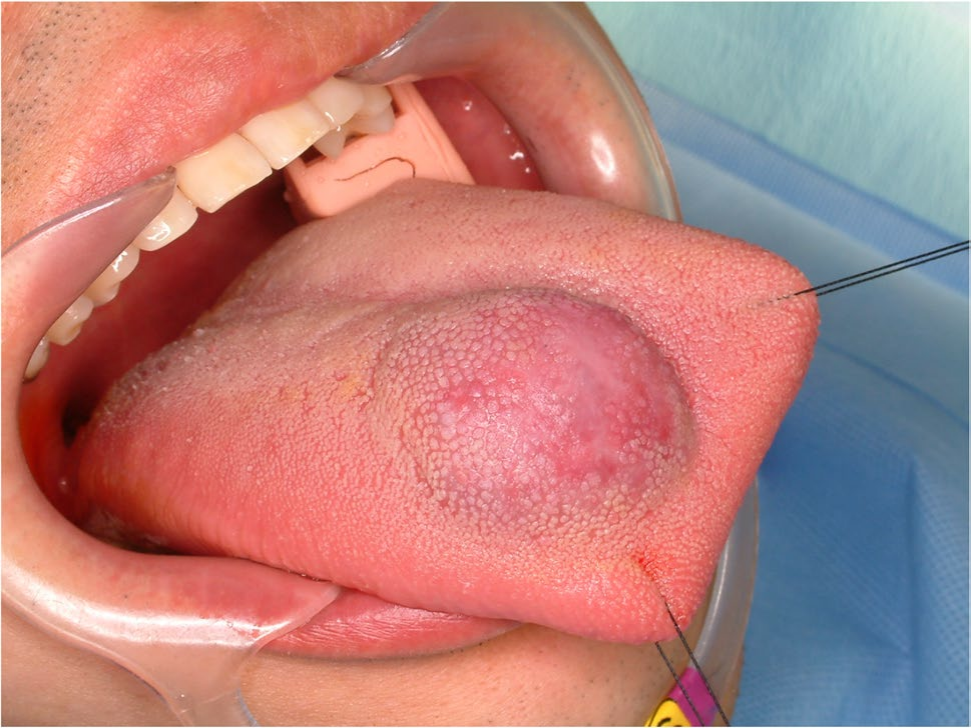
Hemangioma of the tongue

**Fig. 2:**
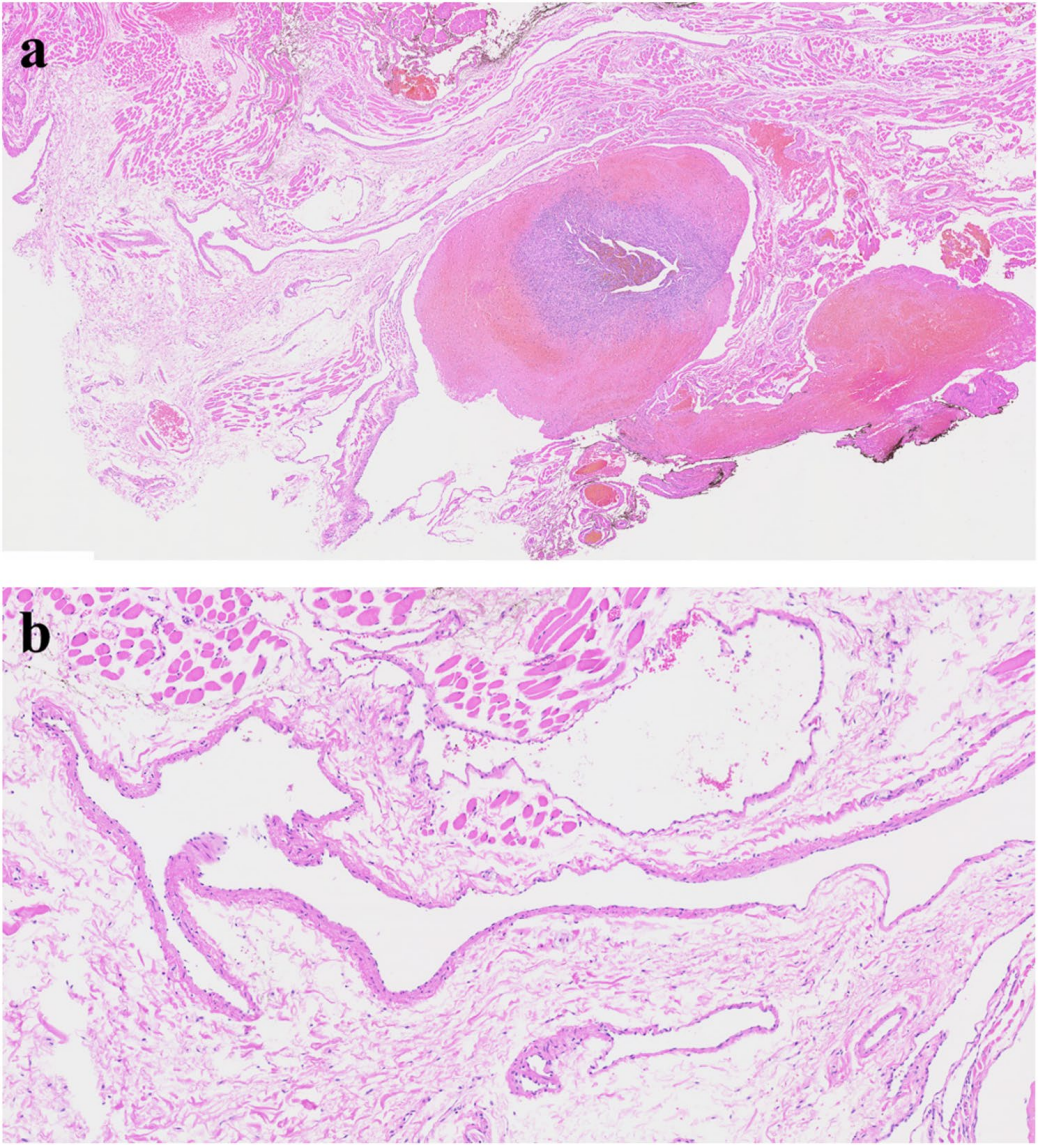
Histopathologic images of hemangioma. (**a**) hemangioma containing intralesional hematoma, (**b**) ectatic capillary and benign endothelial cells of hemangioma

#### Lymphangioma

Lymphangioma is quite common congenital malformation of the lymphatics which is present at birth. Lymphangioma of the tongue is relatively rare compared with other parts of the lesions [9]. Large size of lymphangioma of the tongue may cause airway problem, dysphasia and malocclusion. Congenital lymphangioma of neck area is called cystic hygroma. If the airway is compromised, tracheostomy is mandatory [10]. The differential diagnosis of tongue lymphangioma is relatively easy because of the less vascularity of the lesions compared with hemangioma. The treatment options are same with hemangioma. Sclerosing agents with OK-432 is effective in huge size of lymphangioma [11]. Recently, combined injection of the triamcinolone, bleomycin, and bevacizumab which is effective inhibitor of vascular endothelial growth factor had been tried with successful result [12].

#### Tongue varices (Fig. 3)

Tongue varices are usually located in the posterior-lateral part of the tongue [13]. The most common sign for tongue varices is bleeding. When patients report frequent bleeding without any periodontal disease and ulcer, tongue varices should be considered. Treatment of varices are simple excision and suture or electric coagulation. Usually there is no recurrence after complete excision.

**Fig. 3:**
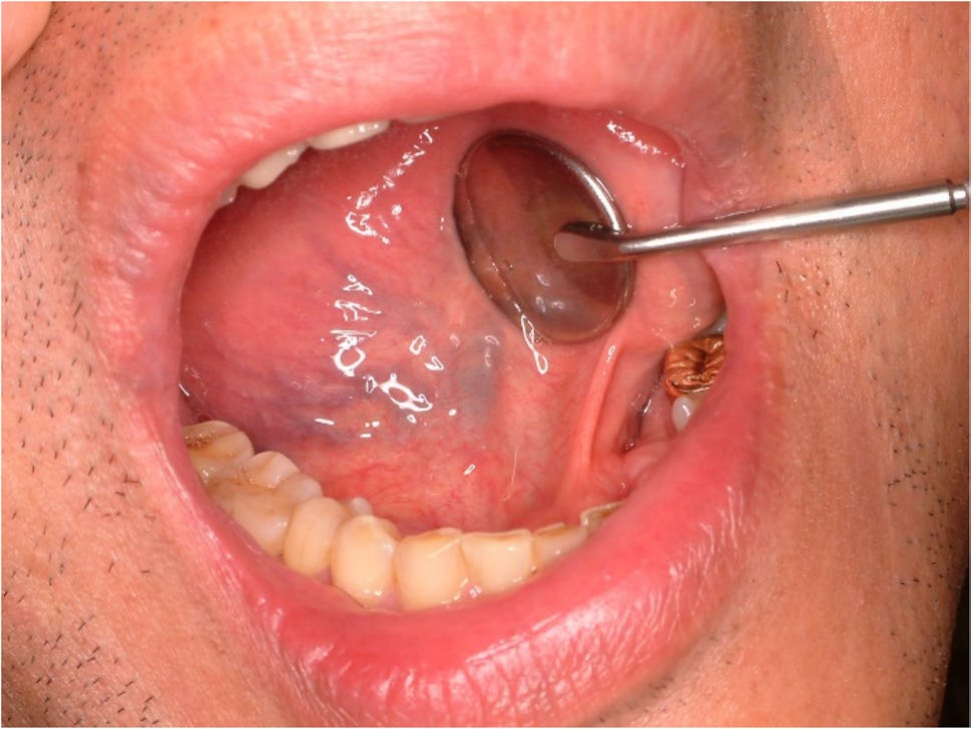
Tongue varices

### Reactive and inflammatory processes

#### Hairy tongue (Fig. 4)

Hairy tongue is a descriptive term referring to an elongation of the filiform papillae because of late exfoliation of the epithelium. Suggested etiologies for hairy tongue is poor oral hygiene, nutritional deficiency and xerostomia [14]. Hairy tongue is often found in heavy smokers and candida albicans infection. Usually, patients are asymptomatic and found during oral care service or tooth brushing. Because of its benign entity, aggressive treatment is not indicated. Self-regression is often found and conservative treatment with gentle debridement with soft toothbrush. Bad habits such as smoking, alcohol, coffee and tea drinking that could cause hairy tongue should be stopped [14].

**Fig. 4:**
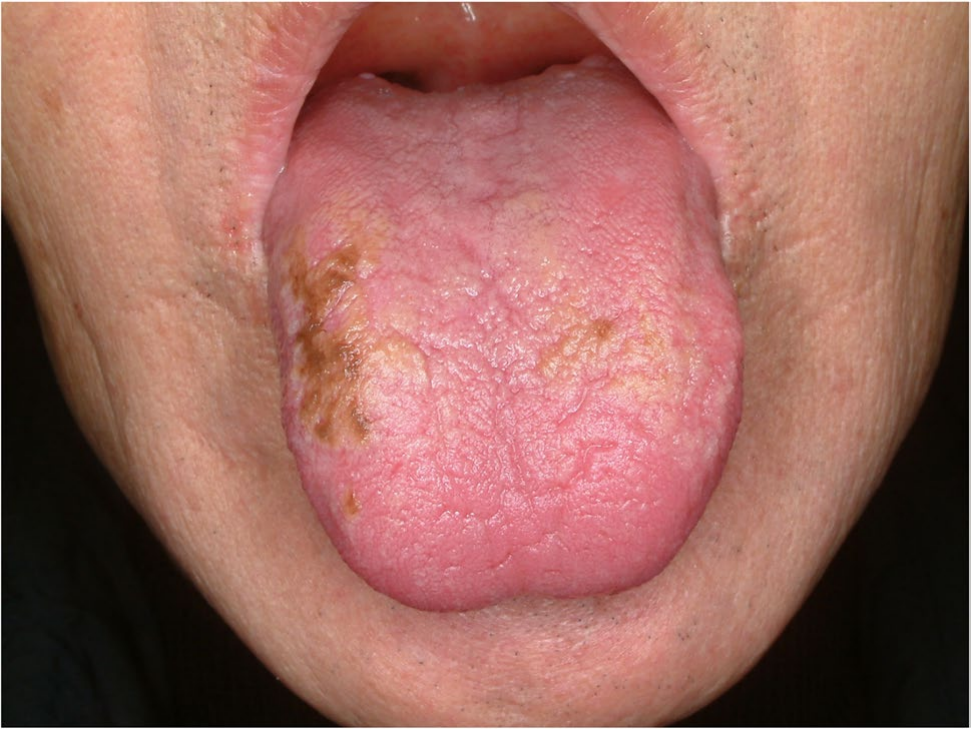
Hairy tongue

#### Pigmentation (melanotic macule) (Fig. 5)

Melanotic macule of the mucosa is commonly found in the oral cavity [15]. Palatal mucosa, lips and attached gingiva are the most common areas. Melanotic macule in the tongue is not common. Most of the melanotic macule presents in multiple lesions, however, solitary lesion is often found. If the shade and size are pale and small, observation of the lesion is recommended. Histologically, the melanotic macule is formed by increased pigmentation of melanocytes in basal cell layer. Differential diagnosis between melanotic macule and melanoma is quite difficult, so excisional biopsy is recommended if the lesion is conspicuous. Local excision is the treatment of choice for local melanotic macule in the tongue and local recurrence seldom happens [16].

**Fig. 5:**
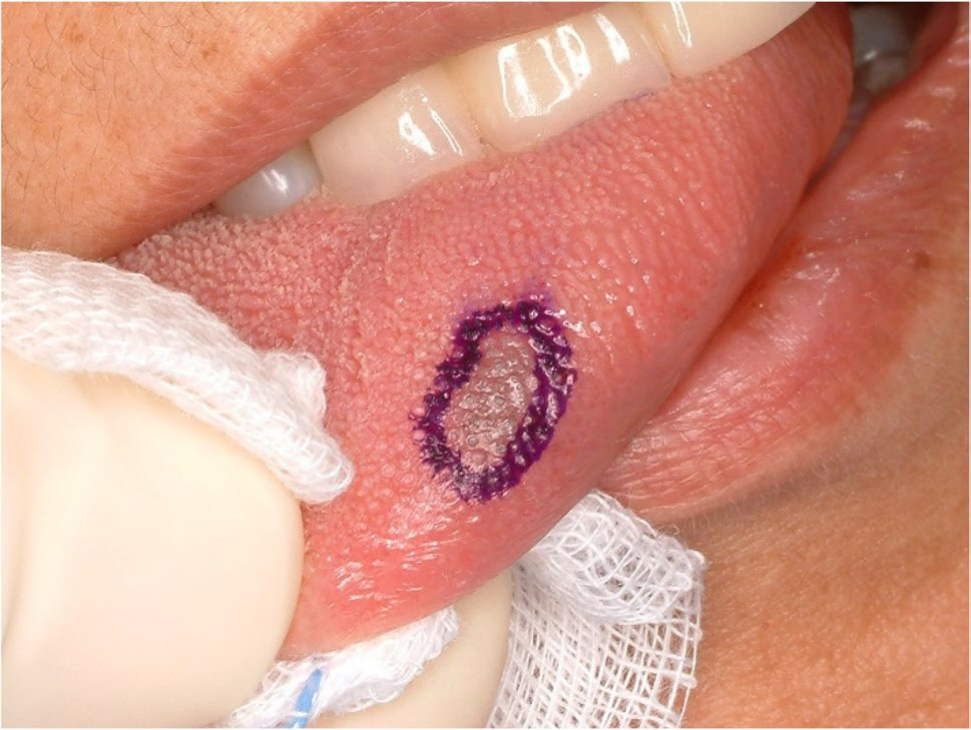
Melanotic macule in the dorsal surface of the tongue

#### Median rhomboid glossitis (Fig. 6)

Median rhomboid glossitis (MRG) is quite common in middle-aged women, and the etiology is not clear. Symptoms include burning sensation, discomfort during eating and swallowing and pain during eating spicy food, however, asymptomatic lesions are often found. MRG is often combined with other tongue disease such as fissured tongue, geographic tongue and candidiasis [17]. The suggested etiology of MRG is autoimmune disease. Self-regression is often found, and symptomatic treatment is recommended. Local application of steroid such as dexamethasone mouthwash, tacrolimus, diphenhydramine and vitamin B are common prescriptions [18].

**Fig. 6:**
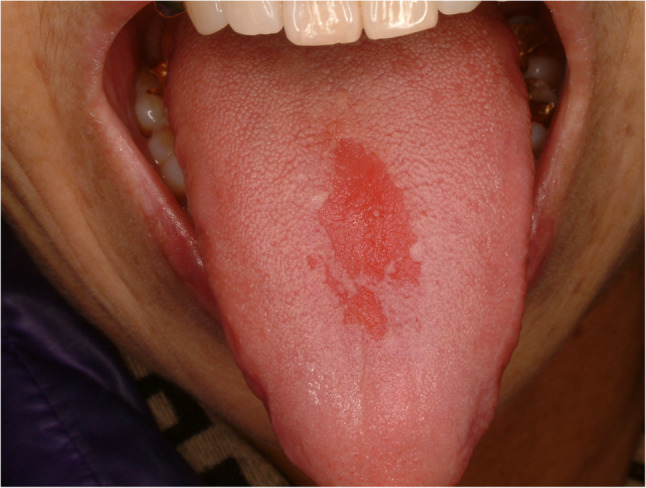
Median rhomboid glossitis (MRG)

#### Fissured tongue (Fig. 7)

Fissured tongue is sometimes a symptomatic condition of painful sensation featured by deep grooves and fissures of the tongue. The prevalence is quite different among studies and symptoms are various [19, 20]. Some contributing factors for fissured tongue are aging, presence of lichen planus, having burning mouth syndrome and smoking [20]. Treatment is focused on reducing symptoms. Topical application of ointment, mouthwashes and oral hygiene care are the most common treatment.

**Fig. 7:**
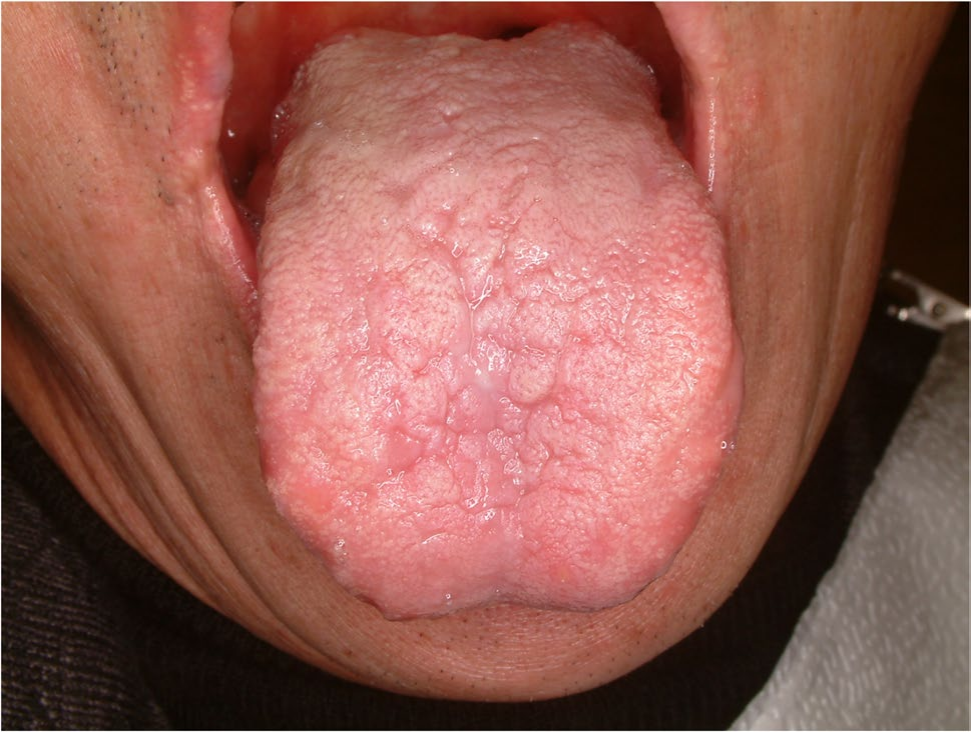
Fissured tongue

### Infectious conditions

#### Oral hairy leukoplakia in AIDS patients (Fig. 8)

Oral hairy leukoplakia of AIDS patients is commonly found in the lateral border of the tongue and is related with opportunistic infection of Epstein-Barr virus (EBV). Oral hairy leukoplakia is an early sign of HIV-induced immune deficiency [21]. Histologically, a thin layer of parakeratosis with positive signals for EBV is found [22]. Ultrastructural examination with electron microscope, herpes-like viral particles were detected in the lateral tongue specimen [23]. Surgical treatment is not recommended for hairy leukoplakia without patient discomfort [24]. Symptomatic treatment with mouthwash, tongue scraper and oral hygiene care is the treatment choice.

**Fig. 8:**
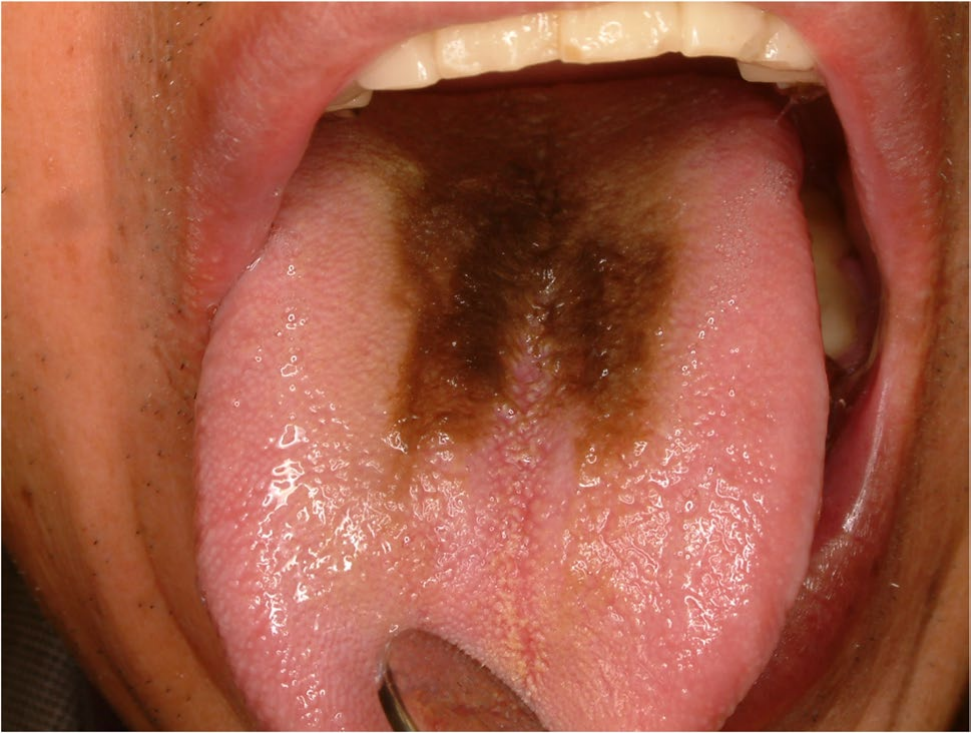
Oral hairy leukoplakia

#### Herpes simplex virus infections (Fig. 9)

Viral infection is world-wide, and herpes simplex virus infection is quite common in all ages. The most common symptoms are redness, painful ulceration and swelling [25]. Usually, viral infection is self-limited and symptoms might be resolved two weeks after initial clinical manifestation. Topical and systemic drugs are indicated when patients complain about the pain and discomfort. Systemic anti-viral therapy consists of Acyclovir (200 mg) five times daily for 7 days. Anti-viral drug (Acyclovir) reduces the periods of ulceration, however, anti-viral drug is most effective before vesicle rupture [26].

**Fig. 9:**
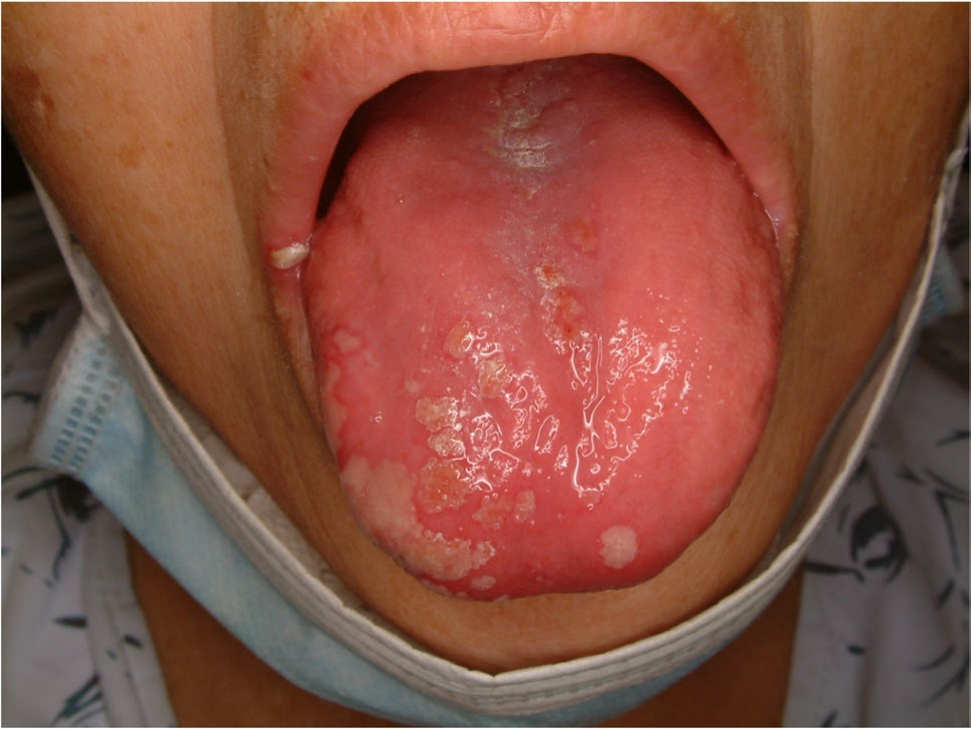
Herpetic stomatitis

#### Squamous papilloma (Fig. 10)

Oral squamous papilloma is commonly seen in the oral cavity and tongue and usually has a benign tendency. The lesion showed exophytic papillary architecture with benign squamous epithelial hyperplasia, parakeratosis or orthokeratosis, and well-formed fibrovascular cores without dysplasia (Fig. 11). Squamous papilloma is associated with the proliferation of human papilloma virus [27]. Whether the papilloma virus could be an etiology for oral squamous cell carcinoma or not is not clear, however, there is some study which reported malignant transformation into squamous cell carcinoma in immunocompromised patient [28]. The early stage of oral squamous cell carcinoma resembles squamous papilloma, especially exophytic type. Early excision, close observation and precise diagnosis with molecular device are required.

**Fig. 10:**
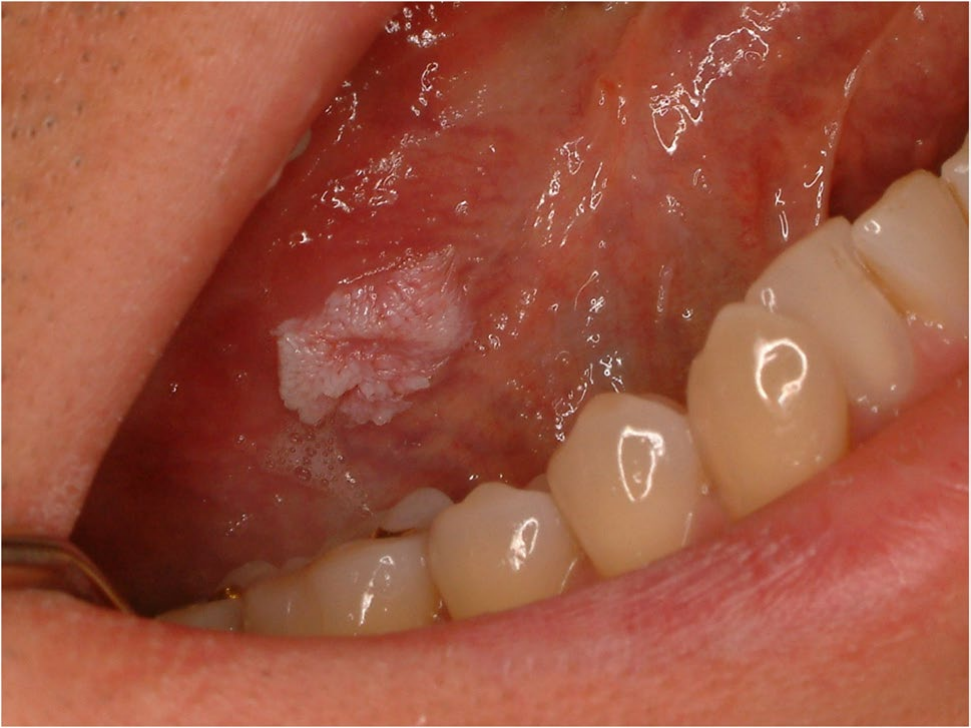
Squamous papilloma of the lateral tongue

**Fig. 11:**
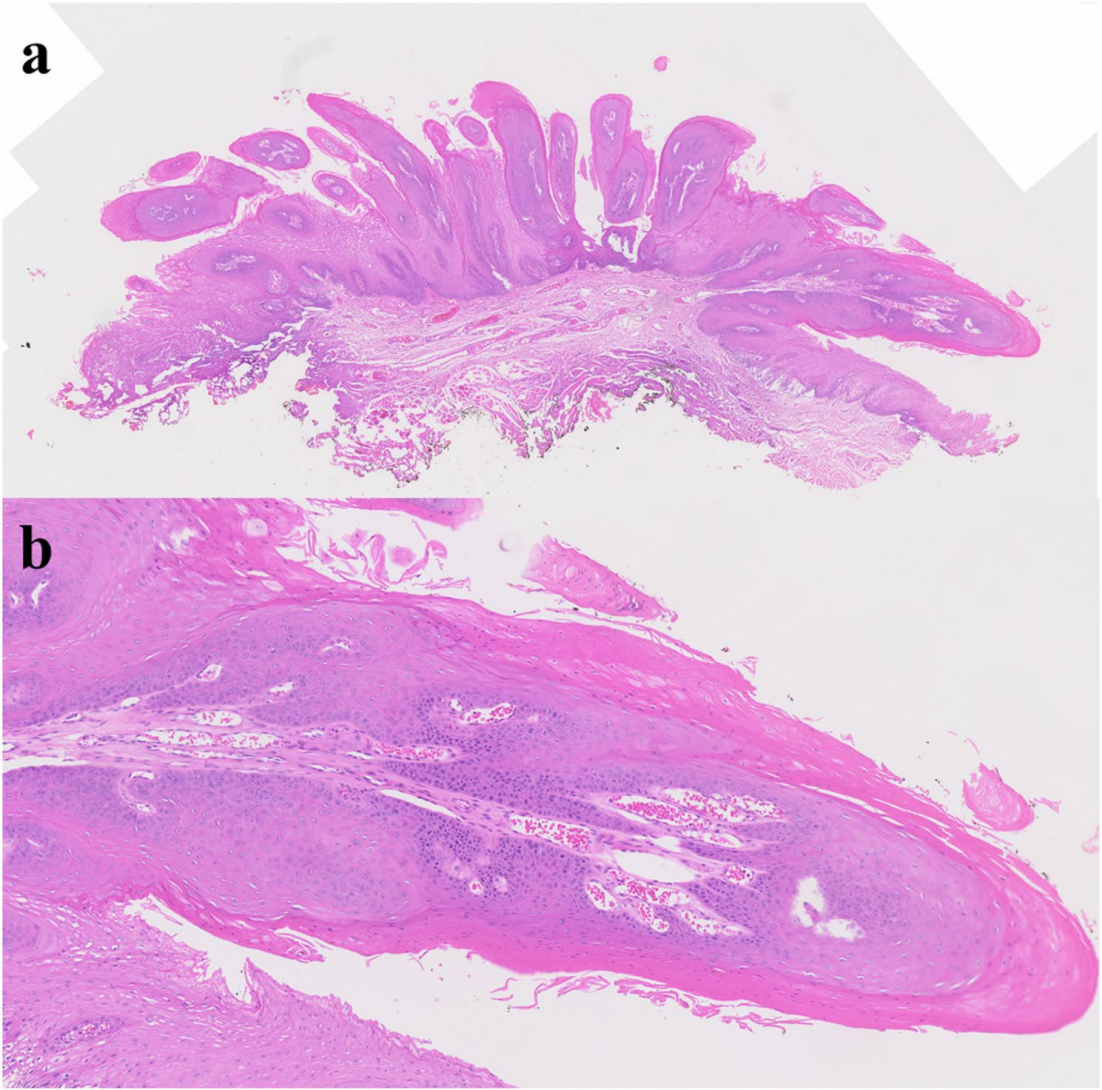
Histopathologic images of squamous papilloma. (**a**) multiple exophytic, finger-like papillary projections at low magnification, (**b**) a narrow branching core of fibrovascular connective tissue with dilated capillaries in a benign squamous cell proliferation

#### Candidiasis (Fig. 12)

Candida albicans infection is frequently found in patients with poor oral hygiene or immunosuppressed condition. Also, it is quite common in patients who wear dentures. It is opportunistic fungal infection by commensal Candida species. Candidiasis in the tongue present with three different type such as pseudomembranous, atrophic, and hyperplastic type. Atrophic candidiasis is hard to differentiate with atrophic glossitis. Candida species infiltrate mucosal surface of the oral cavity. Treatment for candidiasis is antifungal agent (Nystatin 100,000 U/mL) gargle with a recommended dose of 5 ml used in a “swish and swallow” technique four times daily for 2 ~ 4 weeks and additional photodynamic therapy [29]. In case of refractory candidiasis, systemic administration of antifungal drug is indicated. Systemic antifungal agent with nystatin mouthwash for three times a day could effectively diminished the Candida albicans [30].

**Fig. 12:**
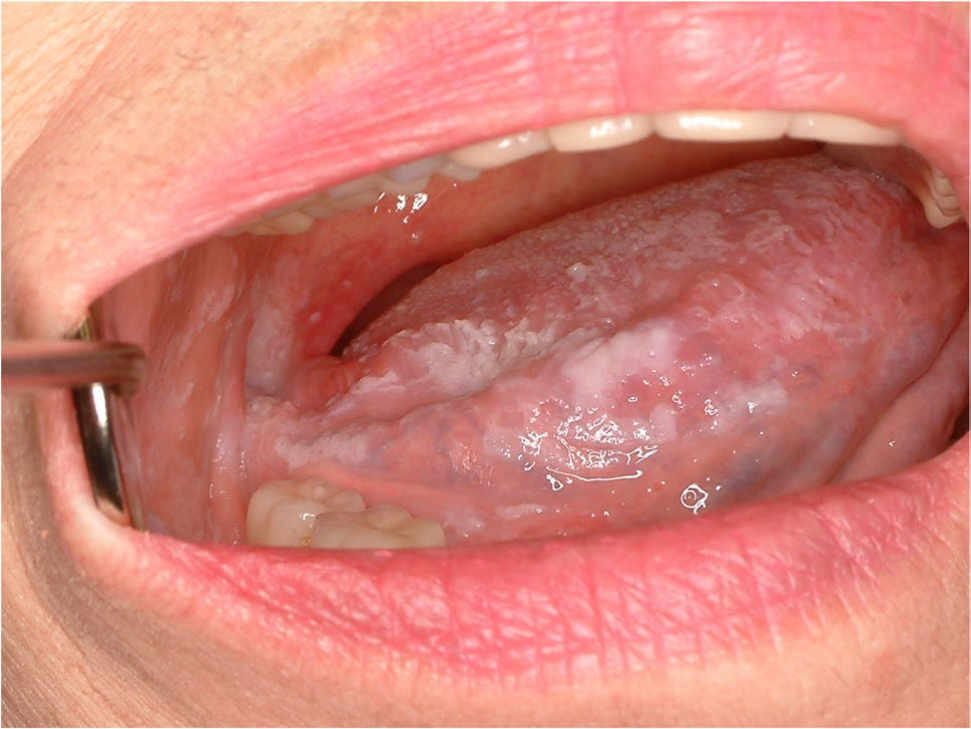
Hyperplastic candidiasis of the oral cavity and tongue

### Premalignant lesions

#### Leukoplakia (Fig. 13)

Leukoplakia refers to a white patch or plaque which cannot be rubbed or scraped off by simple hand pressure. The term of leukoplakia itself is not a disease entity or pathological condition. The malignant transformation of the leukoplakia is known about 5 ~ 20% and recent systemic and meta-analysis showed malignant transformation rate about 19.9% [31–33]. The etiology of oral leukoplakia is diverse, such as smoking, alcohol drinking, inherited, poor oral hygiene, viral infection and immunosuppression. There is an inherited disease characterized by frequent oral leukoplakia such as dyskeratosis congenita which has a defect in DKC1 gene. The triads of dyskeratosis congenita are skin pigmentation, nail dystrophy and oral leukoplakia [34]. Histopathologically, leukoplakia shows loss of polarity with impaired maturation in the lower epithelial layers, increased cellular and nuclear size, and a sharply demarcated transition from the surrounding normal mucosa (Fig. 14). Treatment protocol of leukoplakia is surgical excision. However, if the lesion is too wide and superficial, close observation and stop bad habits are recommended.

**Fig. 13:**
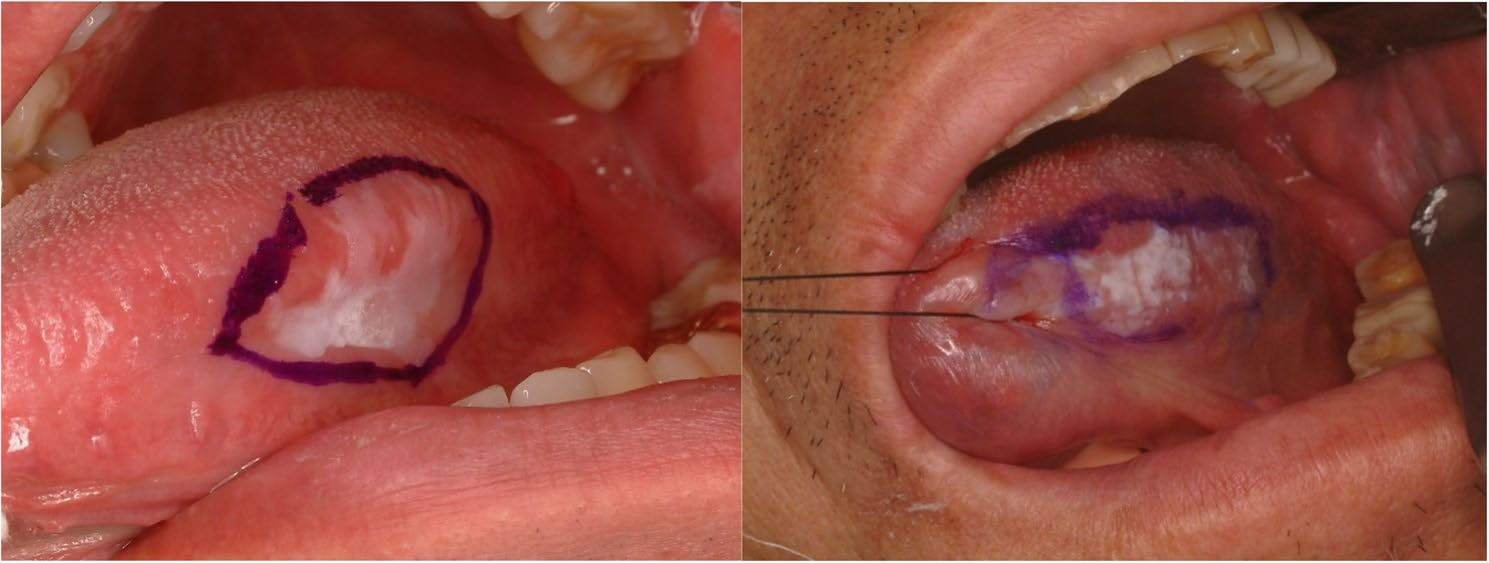
Leukoplakia of the lateral tongue. Local excision and primary closure are recommended

**Fig. 14:**
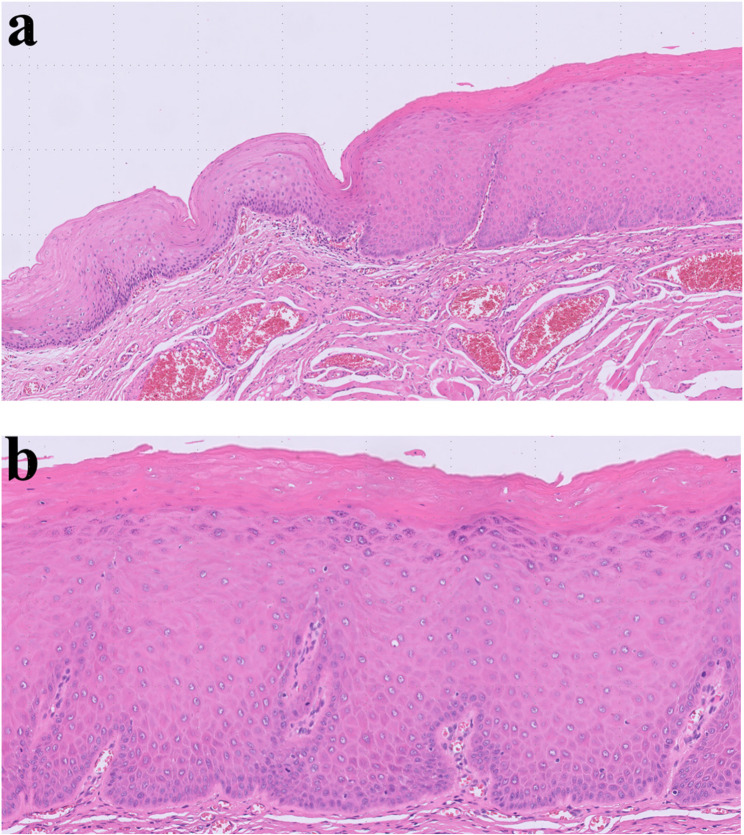
High grade dysplasia. (**a**) normal mucosa in left half, dysplasia in right half, (**b**) atypia in lower half

#### Erythroplakia (Fig. 15)

Erythroplakia is a counterpart of leukoplakia and is also a descriptive term rather than pathologic one. It implies red lesion that cannot be characterized by clinically or pathologically. A definition by exclusion is not scientific and positive description is recommended. A lichenoid lesion which describe a red-colored sharply demarcated lesions has been recommended to substitute the term (Fig. 16.) [35]. Erythroplakia of the tongue is the most common premalignant lesions and potentially 50% of the erythroplakia is transformed into squamous cell carcinoma of the tongue [36, 37]. Most erythroplakia lesions are present with erythroplakia and squamous cell carcinoma at the same time. Treatment protocol for erythroplakia is surgical excision and pathologically precise examination. If the lesion has a carcinoma-in-situ or focal squamous cell carcinoma, wide resection with safety-margin is mandatory. The lesion should be followed at a short interval to watch possible malignant transformation.

**Fig. 15:**
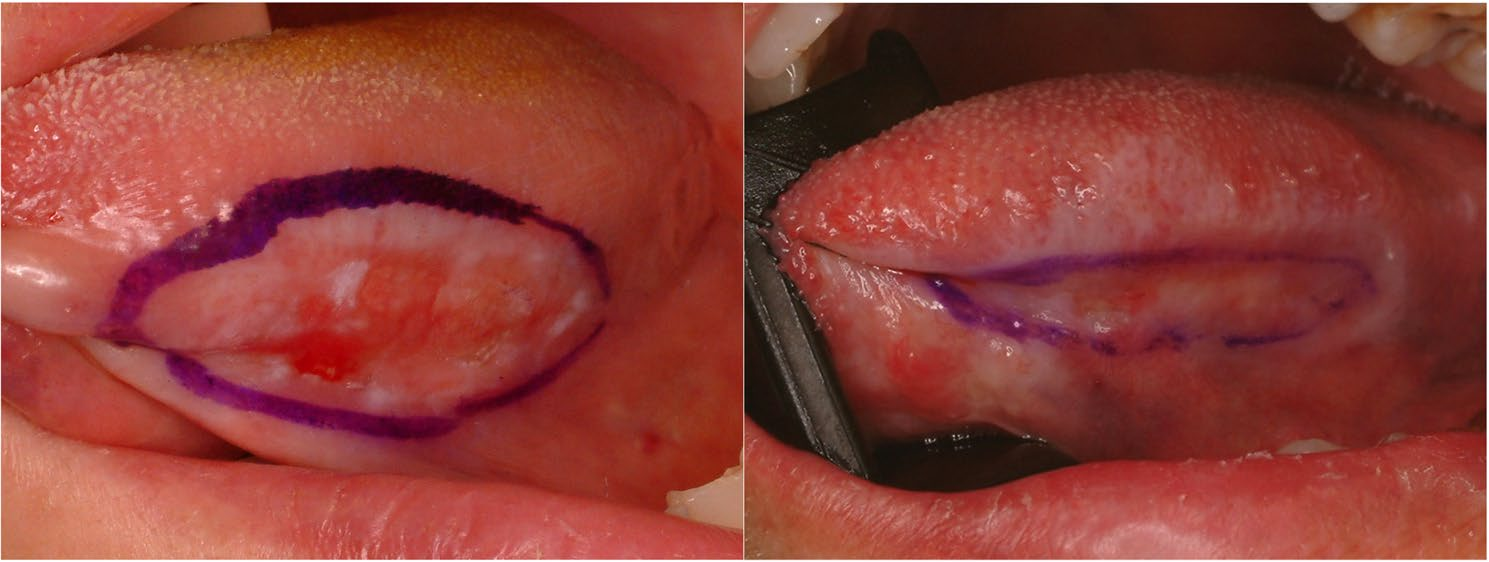
Erythroplakia of the left lateral border of the tongue. Erythroplakia and squamous cell carcinoma are mixed

**Fig. 16:**
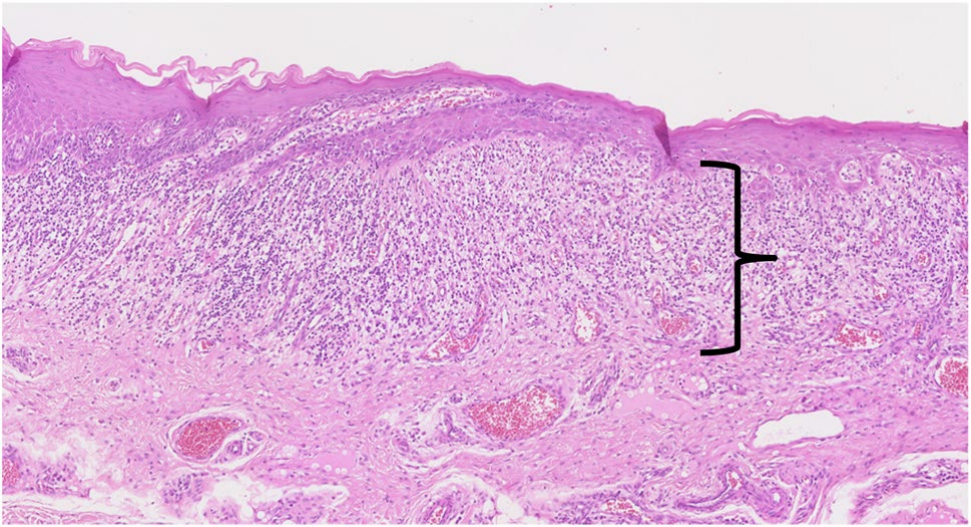
Histopathologic image of erythroplakia (lichenoid glossitis); band-like lymphocyte infiltration, lichenoid reaction

### Benign tumors and cyst

#### Fibroma (Fig. 17)

Fibroma of the tongue is quite common and usually associated with biting habit. Continuous biting of the tongue results in soft tissue scar which results in fibroma. The surface of the fibroma is smooth and shining. Repeated biting of the tongue might cause fibroma, however, spontaneous occurrence without any history of tongue biting is often found. Local excision is enough for fibroma removal and recurrence seldom happens. To rule out the other diseases such as neuroma, Schwannoma and any malignant lesions, postoperative biopsy is necessary [38].

**Fig. 17:**
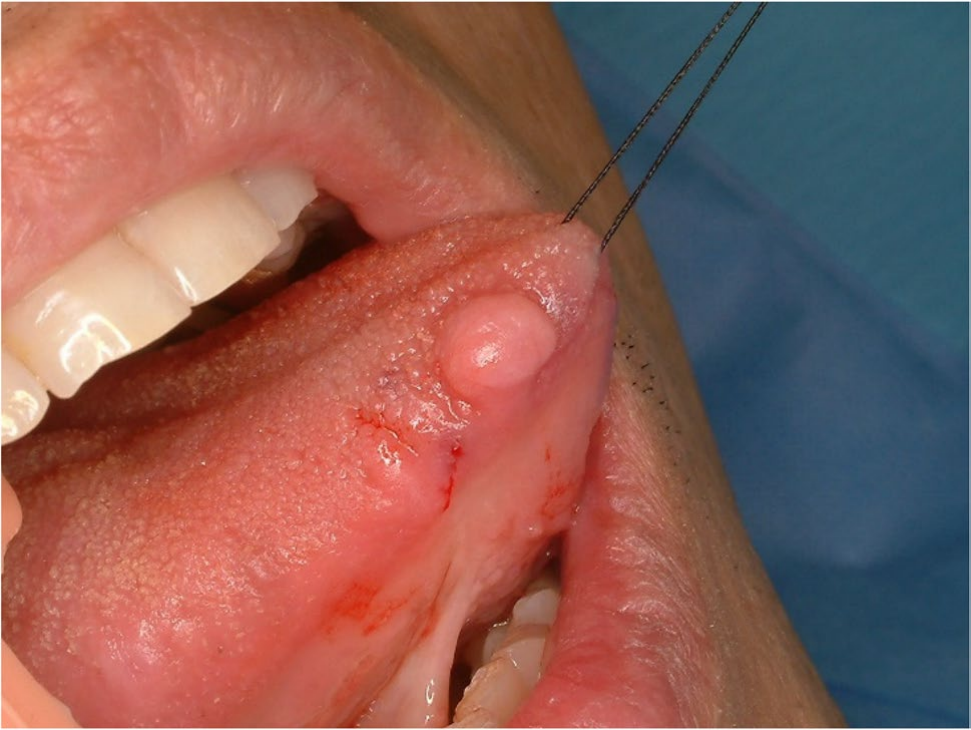
Fibroma of the tongue

#### Lipoma (Figs. 18 and 19)

Lipomas are benign soft tissue tumor which usually occur in the body trunk. The tongue is unusual site for lipoma, and it consists of only 4% of all lipoma in the body. Lipoma in the tongue grows slowly and shows no symptom if the size of the tumor is small. Lipoma in the tongue could be located in intramuscular or superficial. Intramuscular lipoma in the tongue is extremely rare and hard to detect [39]. Magnetic resonance image or sonography could be used to identify the intramuscular lipoma. Histopathologically, the lesion shows features of a neoplasm composed exclusively of mature adipocytes lacking atypia (Fig. 20). Surgical removal is the standard treatment for lipoma in the tongue. Local recurrence is rare if surgical margin is secured.

**Fig. 18:**
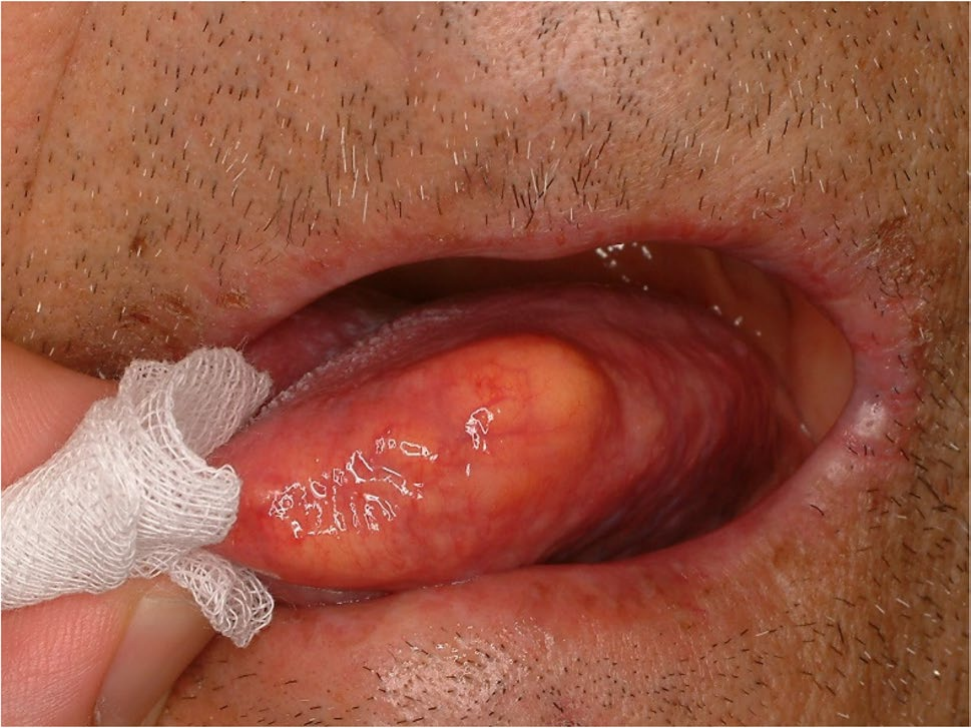
Lipoma of the lateral tongue

**Fig. 19:**
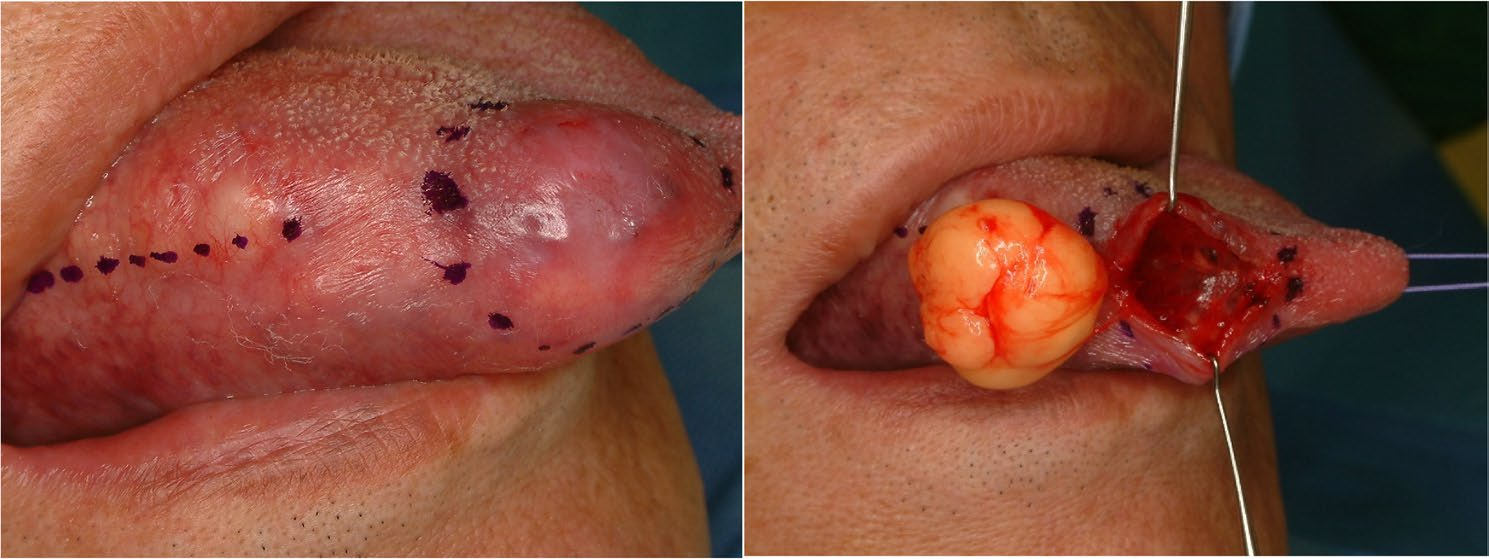
Lipoma of the lateral tongue, that was initially presumed to be a hemangioma

**Fig. 20:**
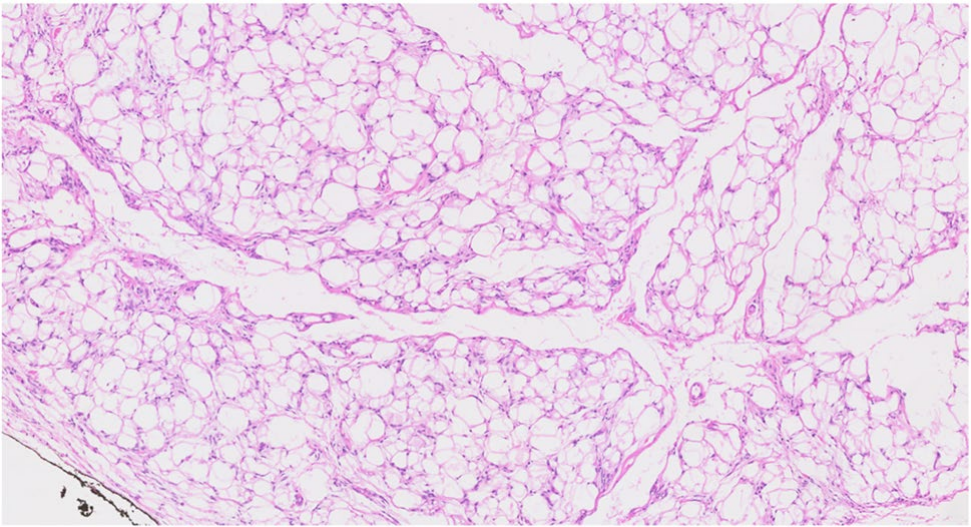
Histopathologic image of lipoma, mature adipocytes

#### Schwannoma

Schwannoma of the tongue is quite uncommon and not many studies were reported in the literatures [40, 41]. There is male predilection and mean age is around mid-30s [42]. Schwannoma is usually located in the anterior mobile tongue and reported no symptom. The unique characteristic of Schwannoma is Antoni A and B areas in histopathology. Surgical excision is recommended, and the lesions is well-encapsulated. If complete removal of the lesions is obtained, there is no recurrence.

#### Rhabdomyoma

Rhabdomyoma of the tongue is very rare and usually found in the base of the tongue. The commonly found symptom is related with dysphasia [43, 44]. This benign tumor is originated from mesenchymal cell from muscles. Even though, the tongue is full of muscles, the incidence of rhabdomyoma is extremely rare. Surgical resection is the standard treatment and recurrence is rare. Malignant counterpart of rhabdomyosarcoma should be differentially diagnosed with excisional biopsy.

#### Neurofibroma (Fig. 21)

Neurofibroma of the tongue is usually found in patient with neurofibromatosis [45, 46]. Neurofibromatosis type 1 is an autosomal dominant hereditary disease characterized by multiple neurofibroma in the whole body. The boundary of neurofibromatosis is not clear with surrounding tissue; it is hard to demark the surgical margin. Solitary lesion of the tongue unassociated with neurofibromatosis type I was also reported [47, 48]. The common symptoms are painless swelling of the tongue and biting. Histologically, the lesion is composed of spindle cells in a collagenous myxoid stroma and the cells showed S-100 protein positive. Single lesions unassociated with neurofibromatosis type I seldom showed recurrence. Surgical removal is the standard treatment, however, there is no encapsulation or demarcation of the lesion. Wide excision is recommended to prevent recurrence [49].

**Fig. 21:**
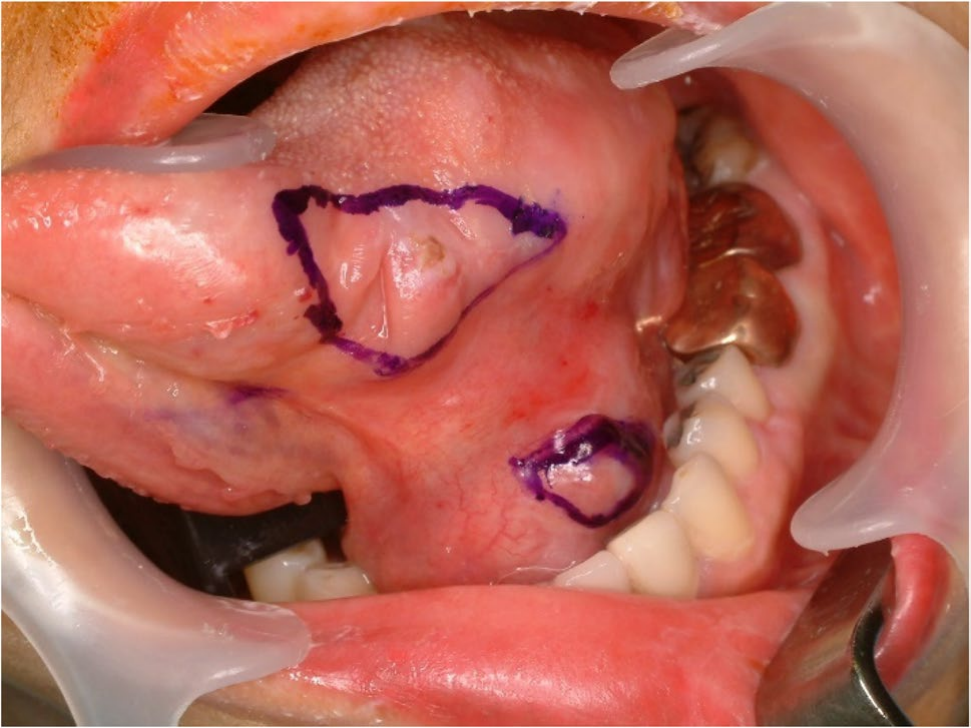
Neurofibroma of the tongue and mouth floor in patient with neurofibromatosis type I

#### Pleomorphic adenoma (Fig. 22)

Pleomorphic adenoma (PA) comes from salivary gland and most commonly occurred in parotid gland. The palate is the most frequent site for minor salivary gland origin PA and followed by upper lips. Tongue is an uncommon site for PA because minor salivary gland is not much populated in the ventral side of the tongue. PA could be found in the dorsal or ventral sides of the tongue and most commonly found in the base of the tongue [50–52]. Pleomorphic adenoma is a well-circumscribed biphasic tumor composed of ductal and myoepithelial cells within a variable myxoid to chondromyxoid stroma and may show focal squamous metaplasia without malignant significance (Fig. 23). Surgical excision without breaking the capsule is the first choice and recurrence is not common. Even though PA is a benign tumor, safety margin with a cuff of normal tissue is recommended [51].

**Fig. 22:**
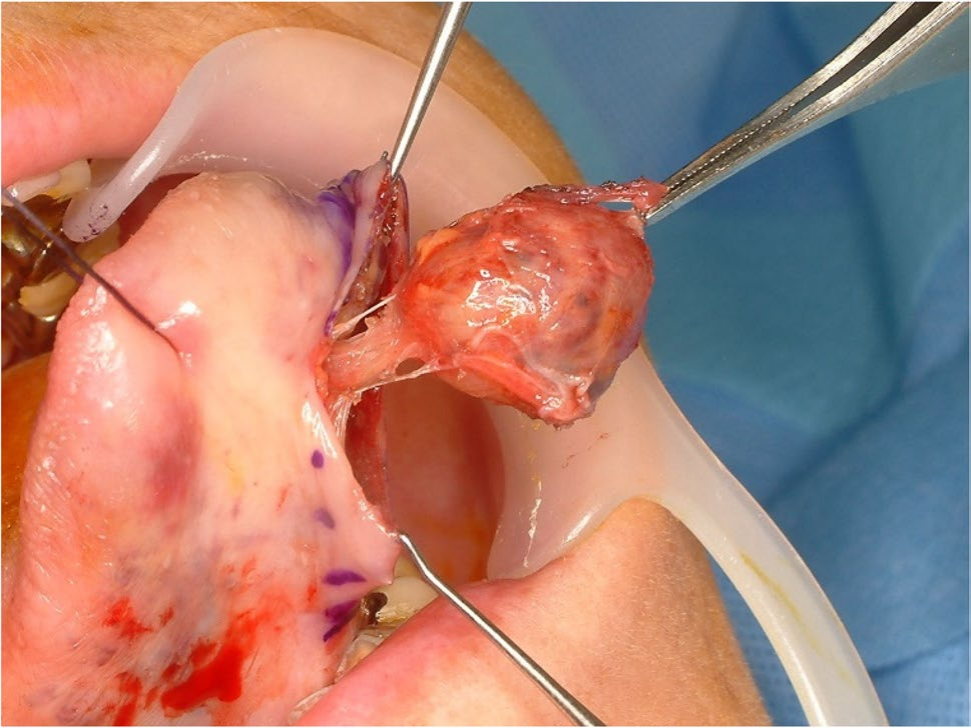
Pleomorphic adenoma of the ventral tongue

**Fig. 23:**
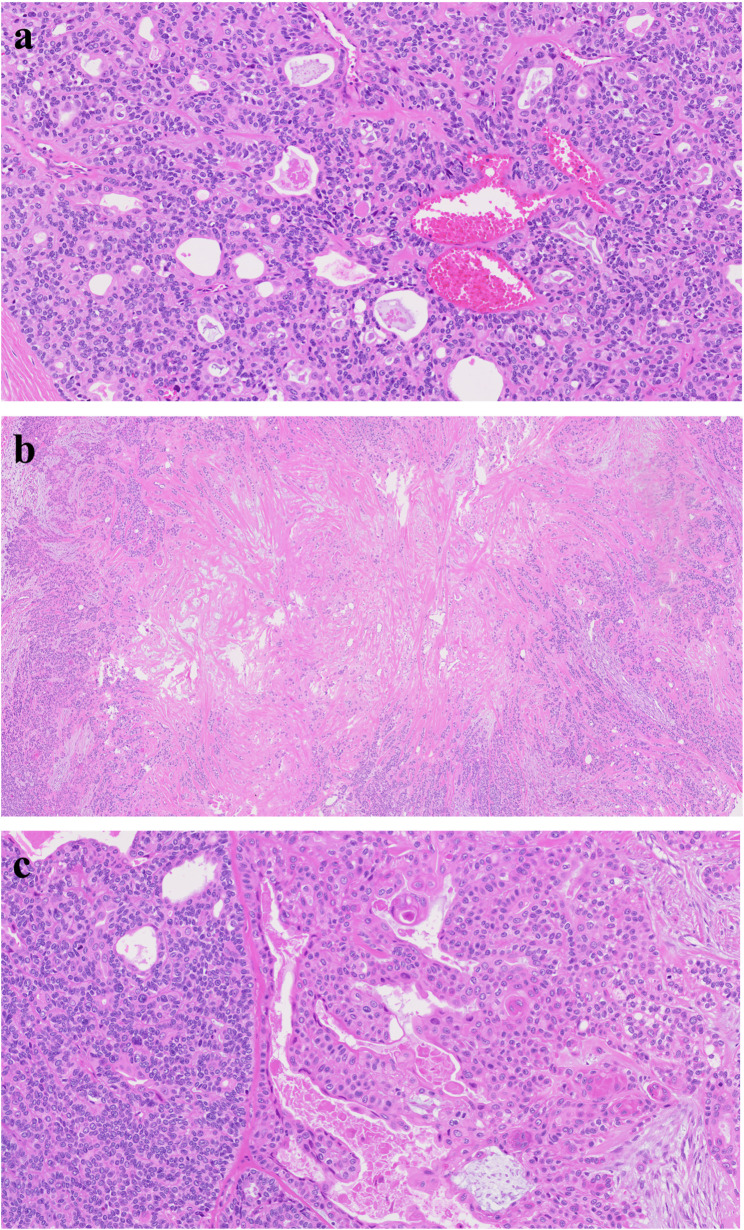
Histopathologic images of pleomorphic adenoma. (**a**) biphasic; ductal cells and myoepithelial cells, (**b**) stromal component, (**c**) squamous metaplasia

#### Myxoma

Myxoma of the tongue is quite rare. The origin of myxoma is mesenchymal cell and myxoma is usually found in the muscles of the hip and lower extremities. Only two intramuscular tongue myxomas were reported in the literatures. Differential diagnosis with other soft tissue tumors is quite difficult. Preoperative biopsy is recommended. Surgical excision is the standard treatment and occurrence was not reported.

#### Dermoid cyst (Figs. 24 and 25)

Dermoid cyst in the oral cavity is quite rare and usually located in the ventral surface of the tongue central area [53, 54]. Mostly dermoid cyst is found in the border between tongue muscle and mouth floor. The histologic characteristics are keratinized squamous epithelium or cylindrical and ciliated epithelium with skin appendages, and fat tissues are commonly found (Fig. 26). Elevated tongue position makes it difficult to swallow and speak. Surgical excision without rupture of the cyst is important. Recurrence is dependent on the surgical margin, so meticulous dissection without damage of the capsule is important [55].

**Fig. 24:**
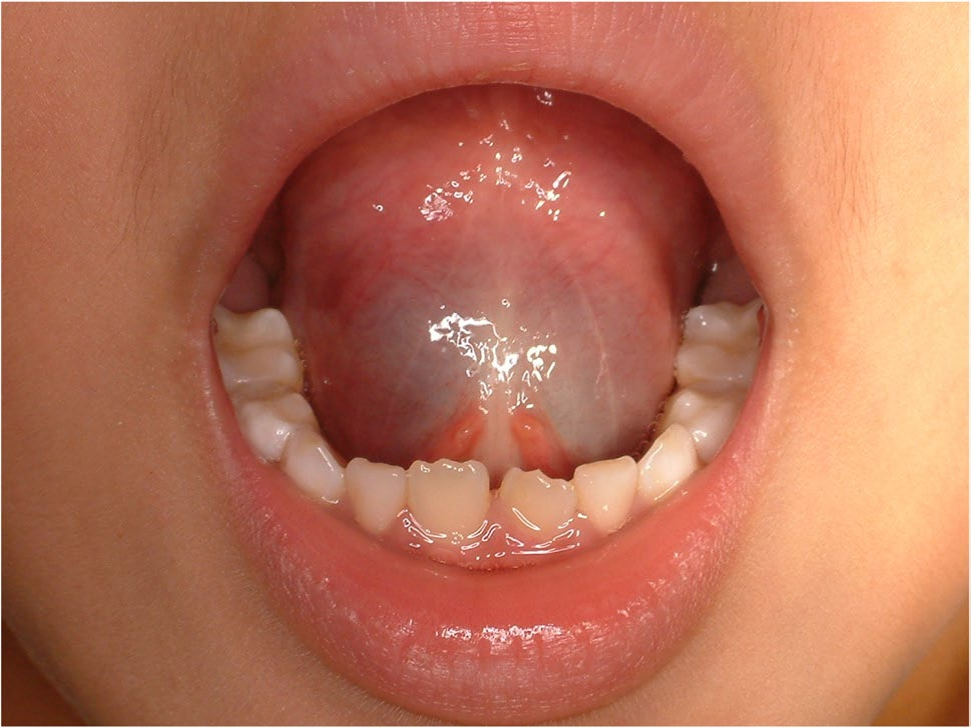
Dermoid cyst in the midline of the ventral tongue

**Fig. 25:**
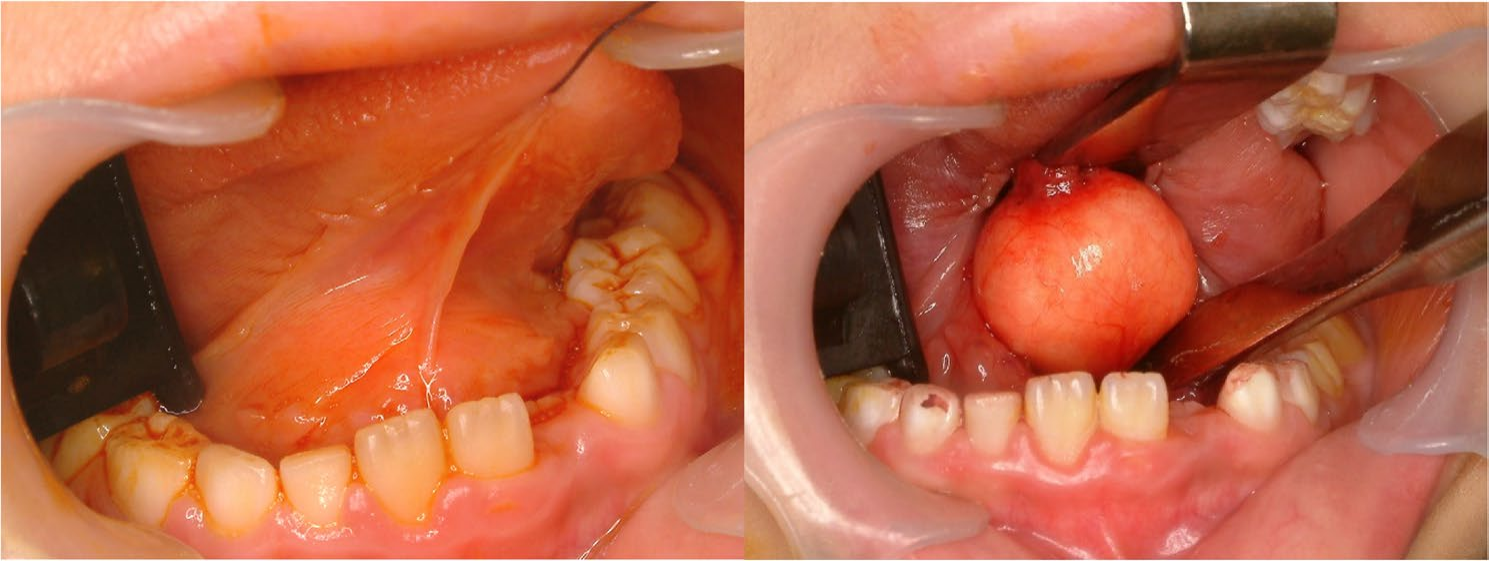
Surgical removal of dermoid cyst in the midline of the ventral tongue

**Fig. 26:**
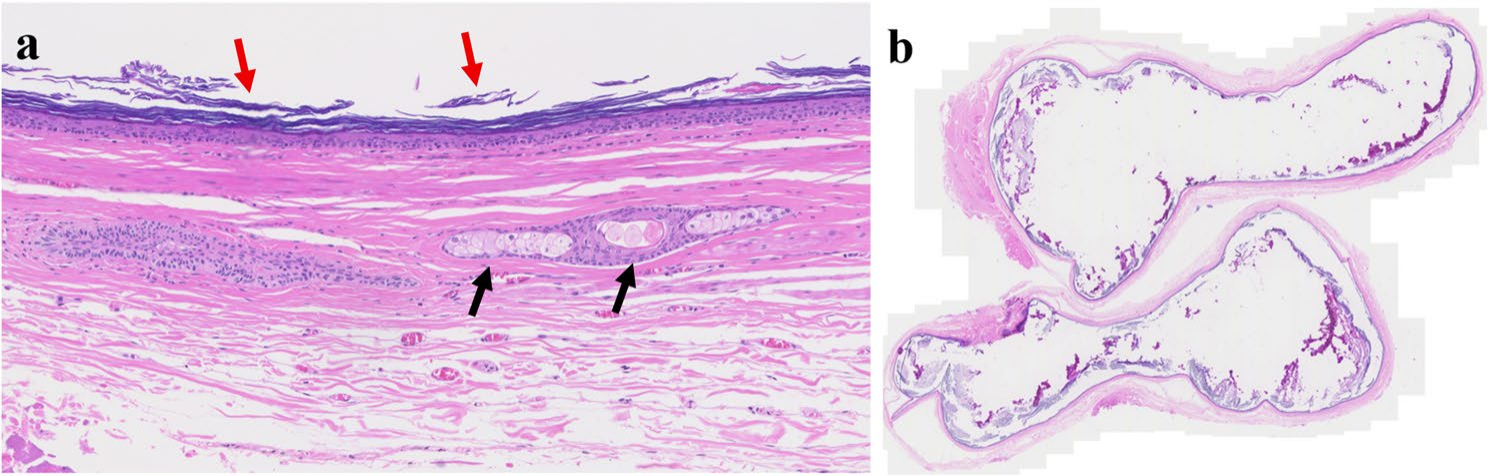
Histopathologic images of dermoid cyst. (**a**) keratin (red arrows) and skin adnexal structures (black arrows), (**b**) dermoid cyst at low magnification

### Malignant tumors

Malignant tumor of the tongue is mostly Squamous cell carcinoma (SCC) and the other entities are quite rare. Oral cavity cancer consists of 5 ~ 7% of all cancer, however, the prevalence and incidence are quite different from each country. In our country, oral cancer comprised of only 1% of all cancer [56]. Among oral cavity cancer, the tongue cancer is the most prevalent in incidence [57]. Differential diagnosis of malignant diseases is quite important because of the lethality of the disease. Most common differential diagnoses are nonspecific ulceration, squamous papilloma, and immune-related ulceration. Exophytic growth of cauliflower shape or endophytic growth with induration.

Surgery is the first treatment of choice for oral cavity cancer. Adjunctive treatment with chemotherapy and radiotherapy is effectively used to prevent recurrence and metastasis. Neck node dissection is required even in N0 neck, because occult metastasis in the tongue cancer is common [58].

#### Squamous cell carcinoma (Fig. 27)

Oral cavity cancer comprises of about 1 ~ 5% of all cancers and the most common site is the tongue and gingiva [57]. Almost all cancers in the tongue is squamous cell carcinoma (SCC) and rarely the other pathology could be found [59]. SCC consists of malignant epithelial cells that show squamous differentiation and infiltrate beneath the basement membrane (Fig. 28). Well-differentiated tumors form large nests and islands with abundant eosinophilic cytoplasm, intercellular bridges, and keratin pearls, whereas high-grade lesions exhibit marked pleomorphism, hyperchromasia, and frequent atypical mitoses with small irregular nests at the invasive front. A desmoplastic stroma with variable inflammation is common, and perineural or lymphovascular invasion may be present in poorly differentiated tumors. Adjacent mucosa may display epithelial dysplasia. Advanced SCC of the tongue has a poor prognosis when regional metastasis is present. Ipsilateral neck node metastasis is common and level I and IIa are the most common sites for metastasis. Neck node dissection and en bloc excision of the cancer are recommended. Surgical removal of cancer is the choice of the treatment, however, advanced stage of the tongue requires multimodal treatment with chemotherapy and radiotherapy. At least 1 cm safety margin is recommended in every aspects of the tongue cancer to ensure a pathological clear margin of at least 5 mm [60]. Partial glossectomy could be primarily closed without any functional deficit. Reconstruction of the resected tongue is mandatory when the defect has unfavorable effects on speech and swallowing. Free flap reconstruction with microvascular surgery is the golden standard [61]. Radial forearm free flap has been the most commonly utilized free flap after glossectomy [61].

**Fig. 27:**
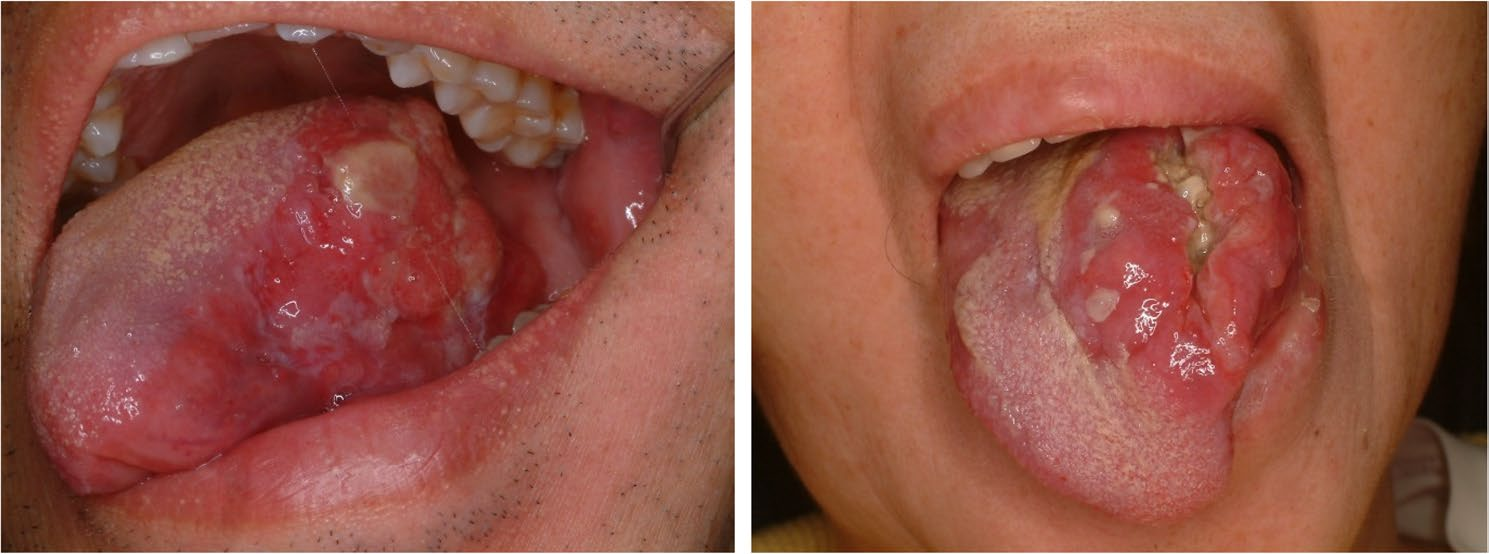
Squamous cell carcinoma on the left tongue

**Fig. 28:**
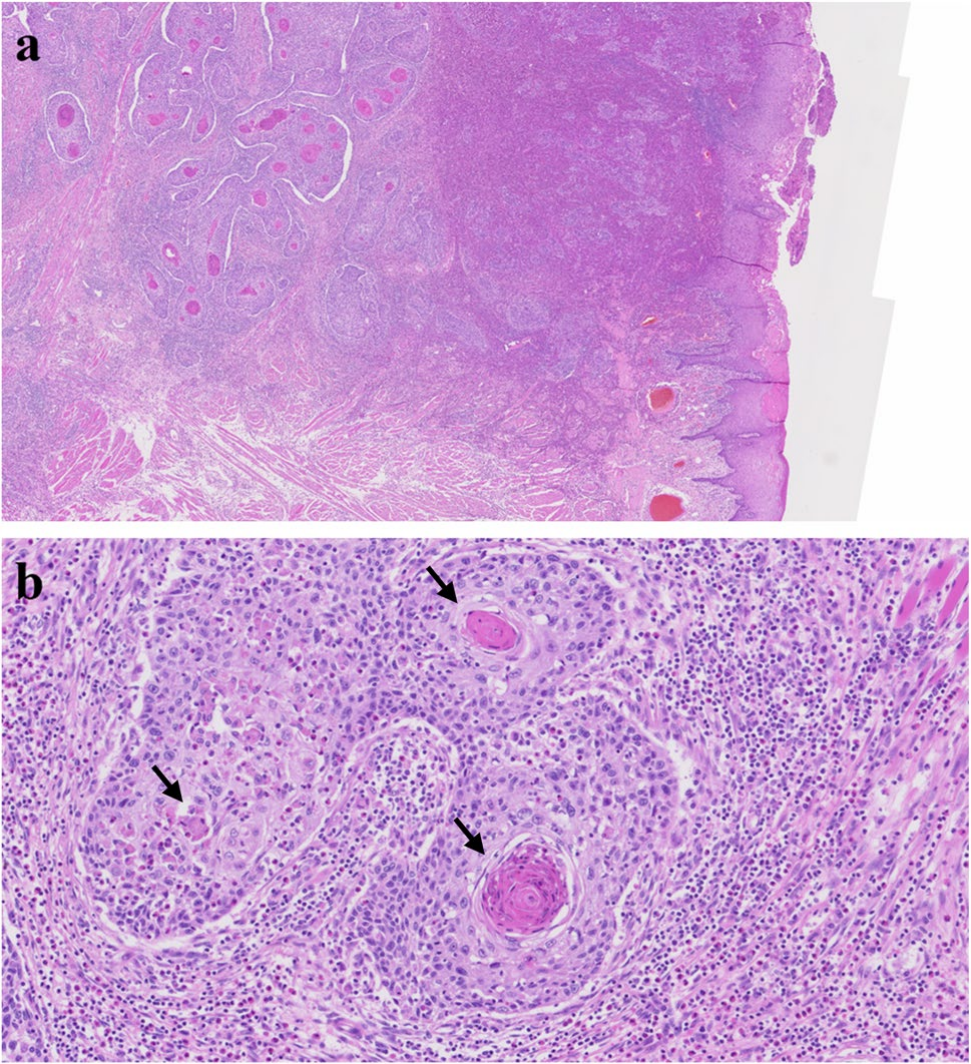
Histopathologic images of squamous cell carcinoma (SCC). (**a**) infiltrative growth, (**b**) atypia and keratin (black arrows)

#### Kaposi sarcoma

Kaposi sarcoma (KS) is often related with AIDS (acquired immune deficiency syndrome) and its occurrence in the oral cavity is typical sign of AIDS related diseases. KS of the tongue is quite rare compared with other parts of the oral cavity [62]. Rarely, KSs are found in non-AIDS and non-immunosuppressed patients [63]. Surgical excision and postoperative radiotherapy are the standard protocol for KS [64]. The prognosis of the KS in AIDS patients are poor and anti-viral treatment and supportive care are important.

#### Oral metastatic cancer from other organs (Fig. 29)

Oral cavity is not a common site for metastasis, however, end-stage of breast cancer, lung cancer and hepatocellular cancer could metastasize to the oral cavity (Fig. 30). Gingiva, mandibular bone marrow, buccal mucosa and maxilla are common sites for metastasis from another organ [65]. The prognosis of the oral metastasis is extremely poor because of the terminal stage of primary cancer. Treatment protocol for metastatic cancer is dependent on the symptom and stage of primary tumor. Multiple organ metastasis with poor systemic health results in poor prognosis and usually does not need any surgical approach for oral cavity mass. However, single metastasis with systemically good condition is indicated for surgical excision. Continuous bleeding and chewing difficulty require palliative surgery for nutritional supply.

**Fig. 29:**
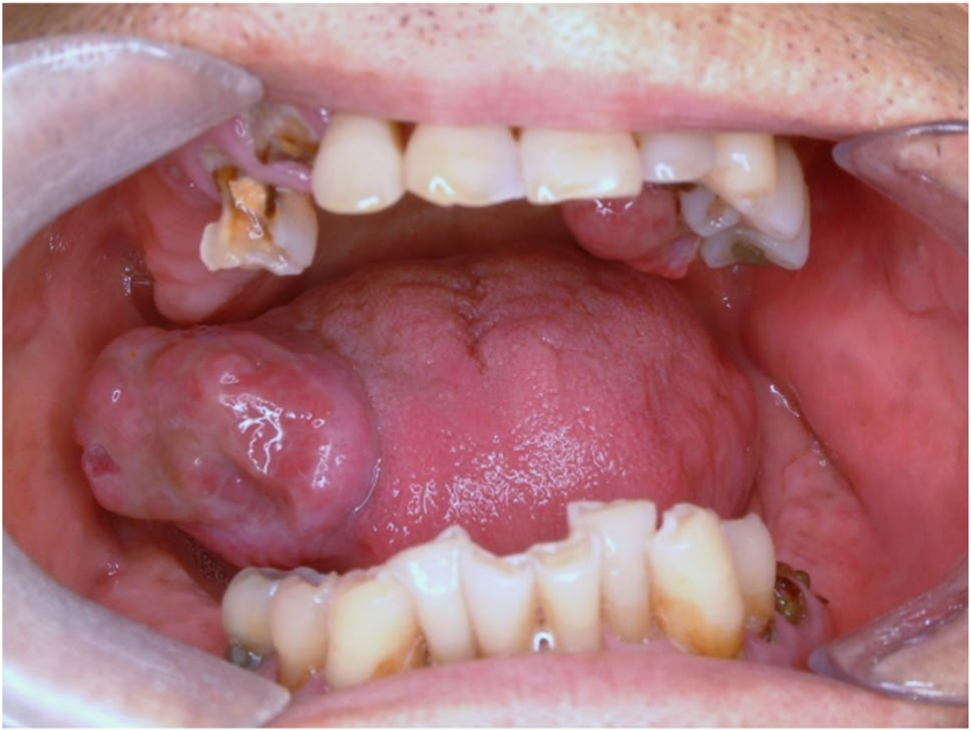
Oral metastasis of the hepatocellular carcinoma in the right tongue

**Fig. 30:**
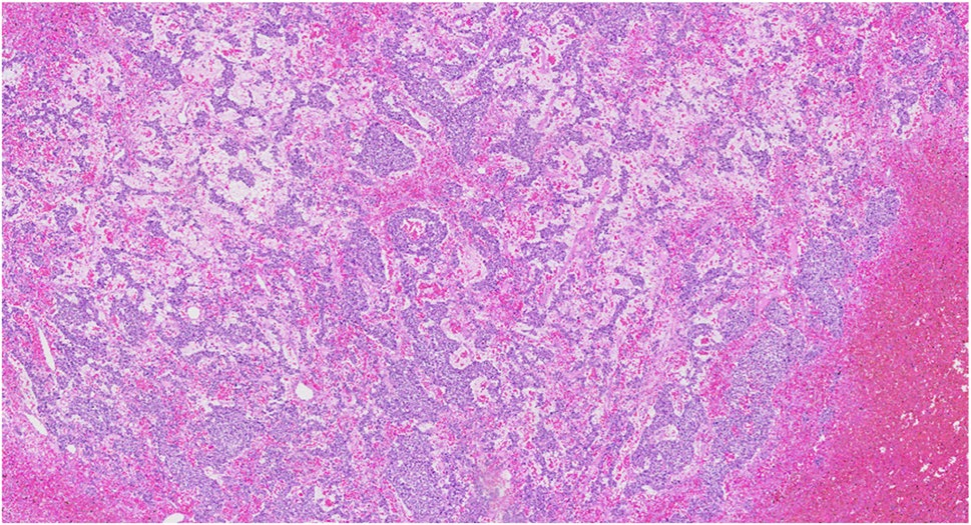
Histopathologic image of metastatic hepatocellular carcinoma; nested pattern

#### Carcinoma ex pleomorphic adenoma

Carcinoma ex pleomorphic adenoma (CXPA) is cancer from pleomorphic adenoma which is the benign counterpart of the CXPA. Tongue is the least common site for CXPA and only one case report was published [66]. Surgical excision is the primary treatment option and radiotherapy has limited effect in CXPA [67].

#### Adenoid cystic carcinoma

Minor salivary gland cancer in the tongue is quite rare, especially adenoid cystic carcinoma (ACC) [68, 69]. ACC of the tongue is common in the base of the tongue and ACC of mobile tongue is extremely rare. Overall survival is poor when lymph node metastasis is present at the time of initial tumor detection [70]. Neck node metastasis is increased in cases of large tumor size, high histologic grade and perineural invasion. Distant metastasis occurs frequently in ACC patients and lungs and brain are the common sites [71]. Wide surgical excision with free flap is the standard protocol for ACC in the tongue.

#### Rhabdomyosarcoma

Rhabdomyosarcoma is the most common malignant tumor originated from soft tissue, especially from skeletal muscle. Oral mass of rhabdomyosarcoma usually originated from parapharyngeal space, and it sometimes fills the whole oral cavity. Oral tongue rhabdomyosarcoma is extremely rare compared with other head and neck area [72]. Complete surgical resection and post-operative chemotherapy are the standard protocol for rhabdomyosarcoma of oral cavity [73].

#### Undifferentiated pleomorphic sarcoma (Fig. 31)

Undifferentiated pleomorphic sarcoma (UPS) is a high-grade soft tissue sarcoma composed of undifferentiated mesenchymal cells with pleomorphic fibrohistiocytic features and no identifiable line of differentiation, making it a diagnosis of exclusion under the current WHO classification (Fig. 32). Historically, UPS was known as pleomorphic malignant fibrous histiocytoma. The involvement of the oral cavity is extremely rare [74]. Complete surgical excision with negative margins remains the primary treatment for localized disease, and neoadjuvant or adjuvant radiotherapy or chemotherapy may be considered [75].

**Fig. 31:**
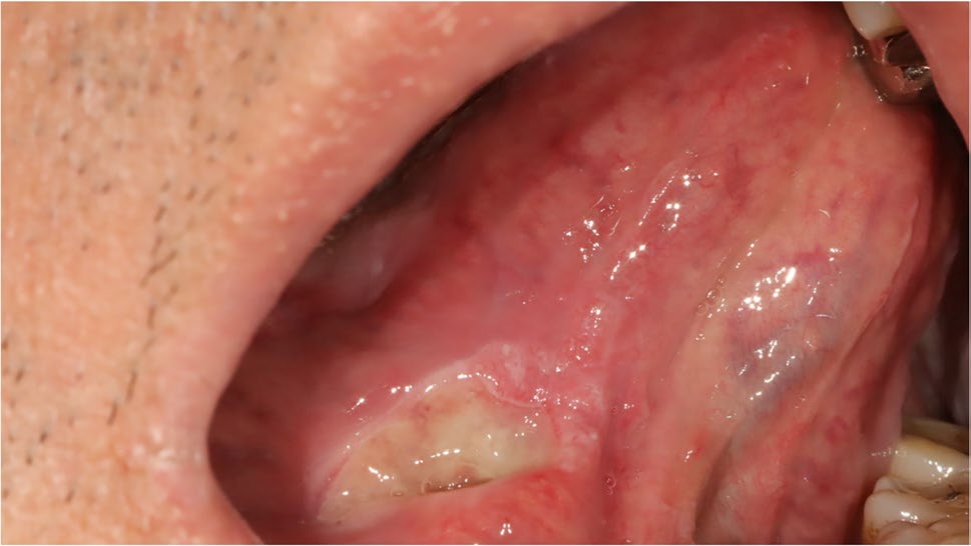
Undifferentiated pleomorphic sarcoma on ventral tongue

**Fig. 32:**
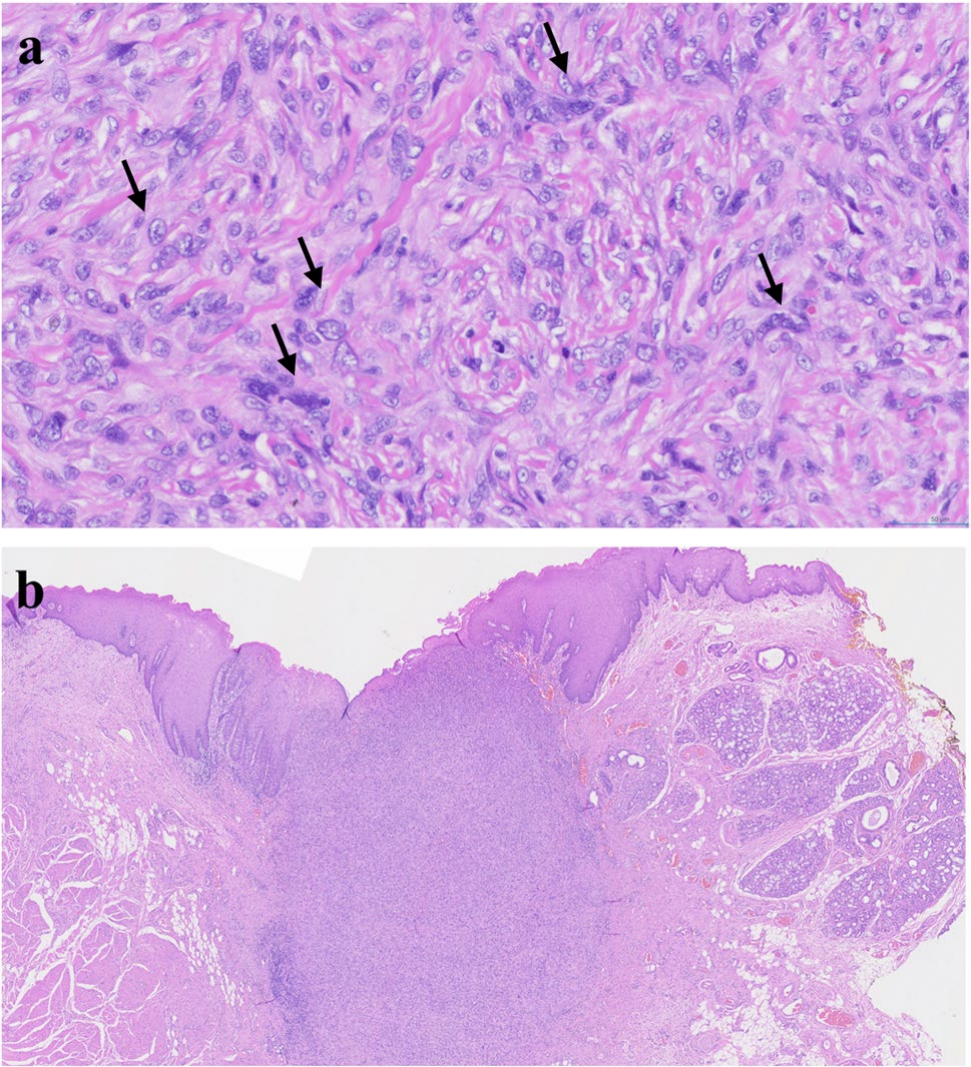
Histopathologic images of undifferentiated pleomorphic sarcoma (UPS). (**a**) mitosis and pleomorphism (black arrows), (**b**) UPS at low magnification

### Symptoms associated with systemic disease

#### Size abnormality -macroglossia (Beckwith-Wiedermann syndrome) (Fig. 33)

Mild macroglossia causes diastema of lower anterior teeth, however, large sized macroglossia could cause airway problem, drooling, snoring, chewing difficulty and dysphasia. The most well-known syndrome associated with macroglossia is Beckwith-Wiedermann syndrome (BWS) [76]. Partial glossectomy is indicated for BWS and design of the partial glossectomy should be individualized for each patient. Lingual artery should be preserved during partial glossectomy.

**Fig. 33:**
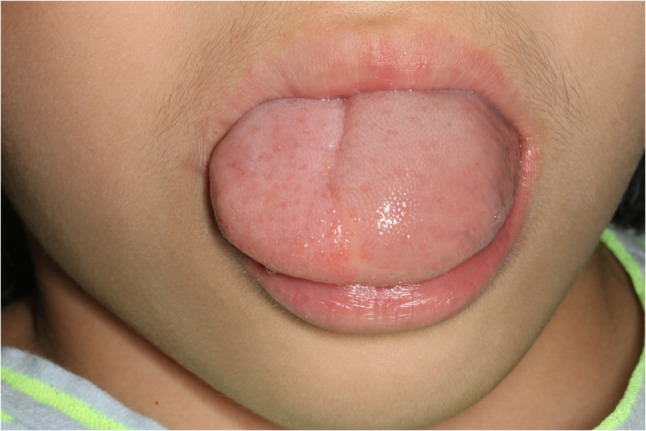
Macroglossia in Beckwith-Wiedermann syndrome

#### Movement disorder - hypoglossal nerve dysfunction (Fig. 34)

Hypoglossal nerve is a motor nerve which involves in tongue movement. Direct injury of the hypoglossal nerve may occur during neck dissection or cancer surgery in the neck. Carotid body tumor of paraganglioma is very rare tumor in the neck which give rise to complications of hypoglossal nerve dysfunction during operation [77]. Direct injury or severing of the hypoglossal nerve results in restriction of the tongue movement. Indirect effect of hypoglossal nerve dysfunction could occur in the brain tumor. Central nerve dysfunction due to brain tumor is common complication and it usually occurred by tumor mass compression. Direct injury of the hypoglossal nerve could be repaired by nerve graft, however, the effectiveness of the nerve graft requires more clinical studies.

**Fig. 34:**
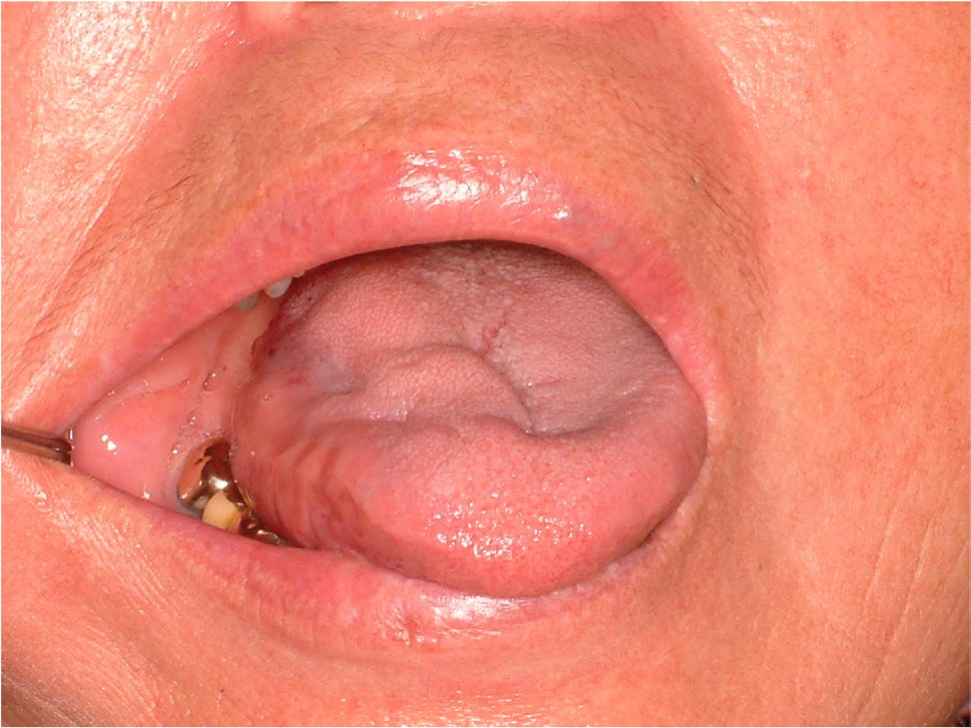
Right side hypoglossal nerve dysfunction due to brain tumor

#### Nutritional deficiency (Fig. 35)

Iron-deficiency anemia (IDA) is one of the most common nutritional deficiencies affecting all ages and races. It is more common in underdeveloped country and children and young generation have higher risk [78]. Serum ferritin level and hemoglobin is usually below normal (women < 12 g/dl, men < 13 g/dl) and it affects various organs. Other laboratory tests such as transferrin saturation, soluble transferrin receptor are recommended to do accurate diagnosis [79]. Tongue is a common involved site in IDA. The clinical sign and symptoms are glossy texture, loss of papilla and burning sensation during eating. Oral iron tablet is prescribed to improve serum ferritin level, however, parenteral iron supplement is more convenient, safe and effective without any gastric trouble [80].

**Fig. 35:**
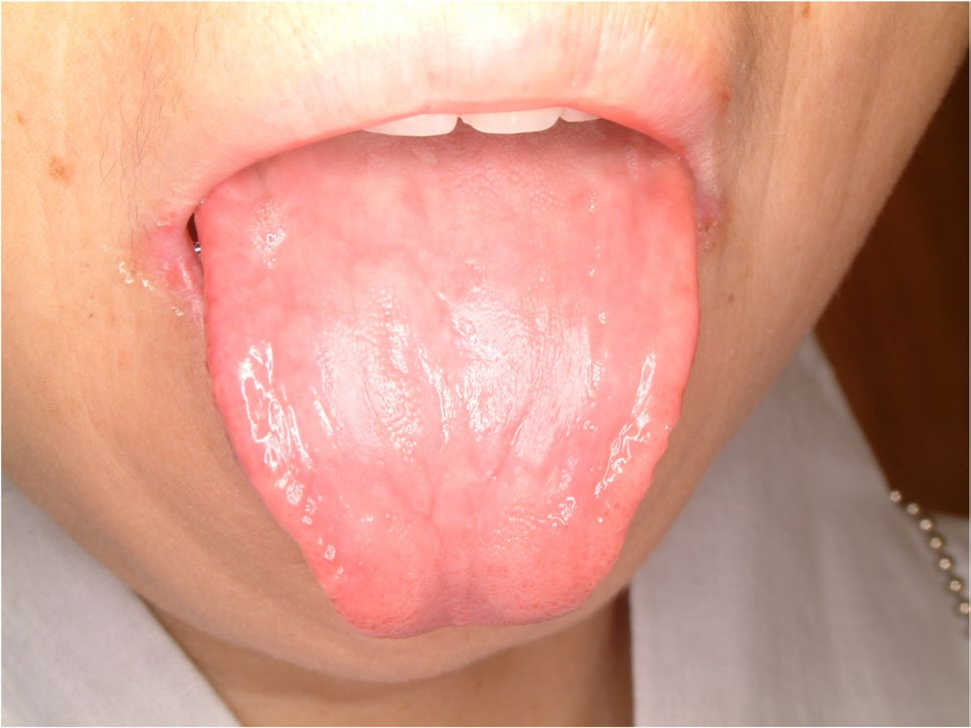
Glossy tongue in iron-deficiency anemia

#### Toxic epidermal necrolysis (Stevens Johnson syndrome) (Fig. 36)

Toxic epidermal necrolysis (TEN) or Stevens Johnson syndrome is an immune related epidermal detachment affecting more than 30% of whole body [81]. The cause of TEN is uncertain, however, adverse drug reaction which was amplified by immune reaction is suggested [82]. The most common causative drugs for TEN are carbamazepine, iopamidol, anti-viral drugs and antibiotics [83]. TEN affects mainly epidermal components, it also involves tongue and mucosa. The mortality rate of TEN is relatively high and it requires emergency treatment. Immediate withdrawal of the associated drug, supportive and nutritional care and dermatologic therapy to prevent hemorrhage are mandatory. The most effective drugs for TEN are cyclosporine, steroids, and prophylactic antibiotics. Low-intensity LASER therapy is recommended to treat skin lesions [84]. Oral lesions are improved by systemic drug therapy, however, topical application of steroid gargle or ointment are recommended to improve ulcer symptoms.

**Fig. 36:**
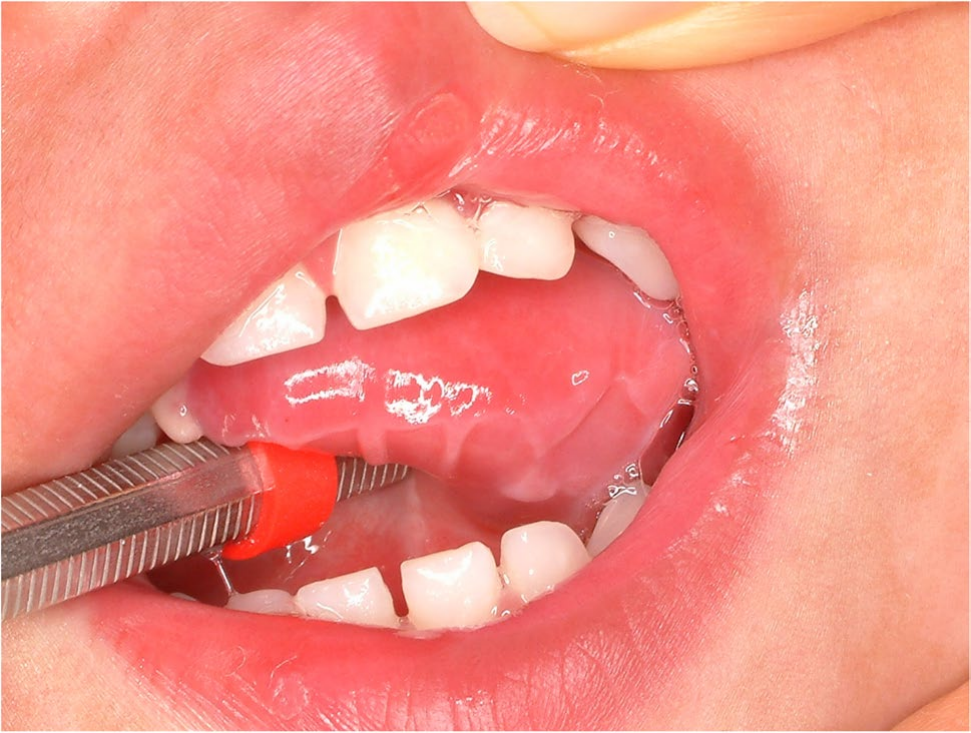
Toxic epidermal necrolysis affecting the lateral tongue

#### Xerostomia (Fig. 37)

Xerostomia or dry mouth of the oral cavity is commonly found in patients who underwent radiotherapy for head and neck cancer, renal failure, diabetes, multi-drug users, and Sjögren syndrome [85]. Xerostomia of the oral cavity is mostly induced by reduced secretion of saliva. The etiologies for reduced salivation is aging, dehydration, anxiety, drugs such as tricyclic antidepressants, and destruction of salivary gland by radiotherapy. One of most devastating cause of xerostomia is Sjögren syndrome which affects eyes, joints, lacrimal and salivary gland. There are two medical treatments for xerostomia. One is artificial saliva [86, 87] and the other is medication which stimulates saliva secretion such as pilocarpine [88]. There are several surgical treatment for xerostomia such as fat grafting [89], autologous mesenchymal stem cell graft [90], and acupuncture [91]. Patient selection and to identify the etiologies should be preceded before selecting optimal treatment.

**Fig. 37:**
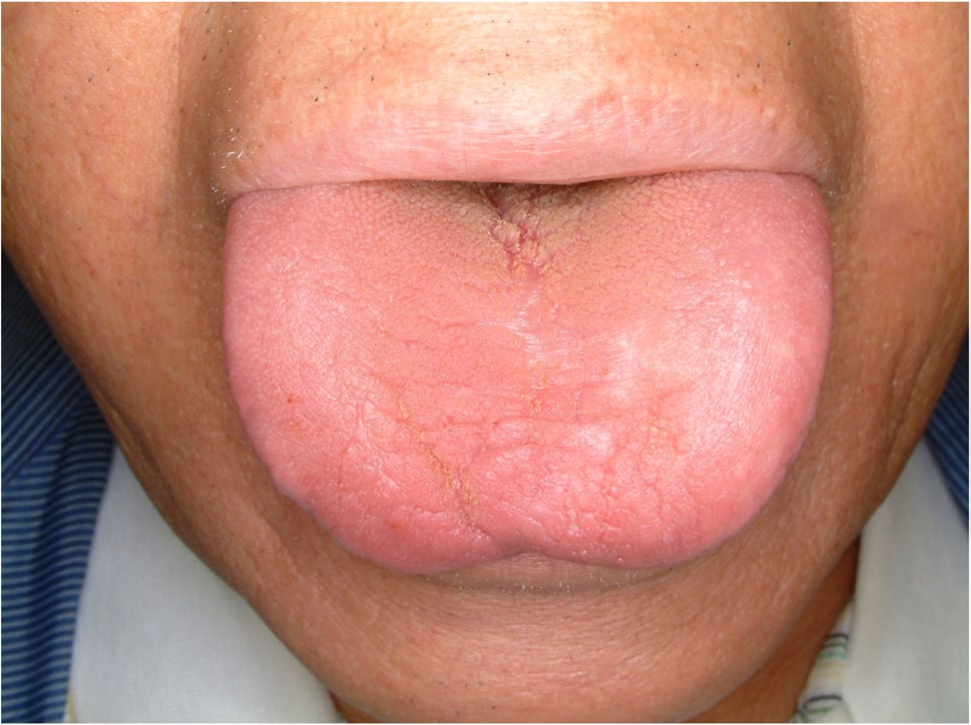
Xerostomia showing dryness of the tongue mucosa and fissure

#### Nonspecific ulcer - geographic tongue (Fig. 38)

Geographic tongue is quite common tongue disease and the prevalence is approximately one in 30 adults [92]. Symptoms of the patients is diverse from no symptom to severe pain and dysphasia. The etiology of geographic tongue is not elucidated, however, autoimmune is suspected. The sign of geographic tongue is erythematous lesions mixed with white patches that resembles map. The lesions could be persisted for several days to several months. Self-remission is commonly found, however, recurrence is quite often. The etiology is not clear and different hypotheses of pathogenesis are suggested [93]. Symptomatic treatment is indicated for geographic tongue. Topical application of steroid gargle, ointment, tacrolimus, pregabalin and low-level LASER therapy are recommended [94–97].

**Fig. 38:**
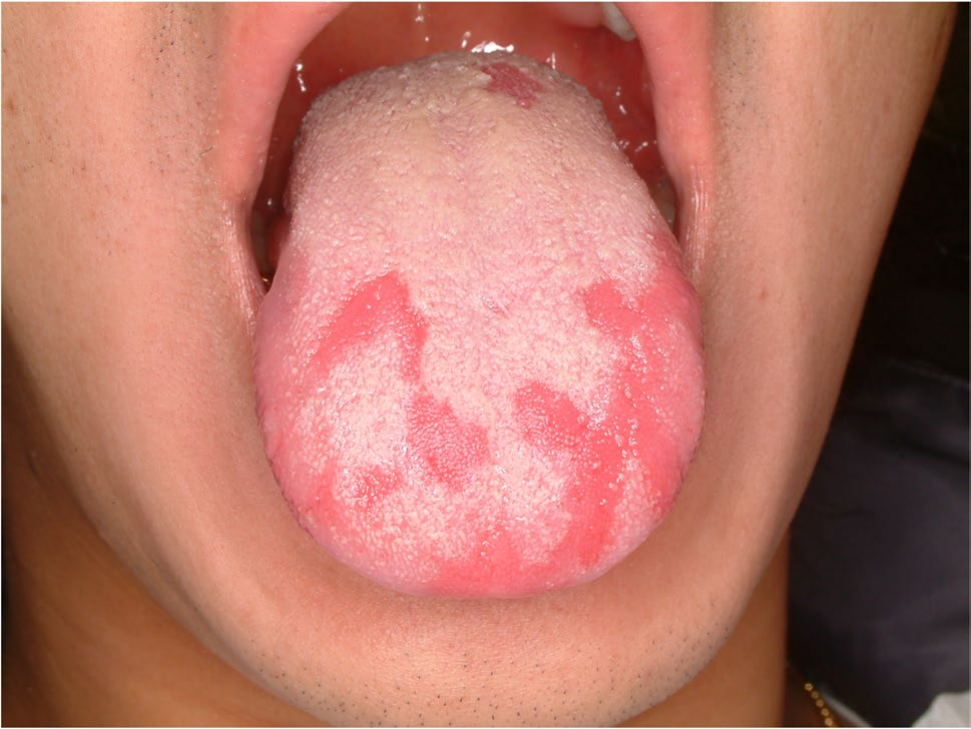
Geographic tongue

####  Sensation disorder - Burning mouth syndrome (Fig. 39)

Burning mouth syndrome (BMS) is an idiopathic, unexplained pain condition characterized by a burning sensation of the oral mucosa in the absence of any specific organic disease [98–100]. There are many hypotheses for BMS mechanism. One is suspected to be related with neuropathic pain from the peripheral or central nervous system. Another condition is hormonal unbalance which is quite common in women and also BMS is. However, the relationship with local or systemic disease and BMS has not been elucidated. BMS may be divided into primary or secondary. Primary BMS does not have any sign of pathology in the tongue with continuous pain. Secondary BMS is related with post-operation sequelae. The treatment of BMS has not been found and usually symptomatic treatment with medication are indicated. Topical application of ointment, gargle or systemic administration of drug relieve symptoms, however, complete remission of discomfort is not common. GABA (gamma-aminobutyric acid) inhibitor, tricyclic antidepressants, selective serotonin reuptake inhibitors and gabapentin are the most commonly prescribed drugs to reduce symptoms. Neuropathic pain is managed by titrating Gabapentin, starting at an initial dose of 300 mg/day and gradually increasing up to 900–1,800 mg/day based on patient response. Topical application of capsaicin, honey-based chamomilla tinc and steroid gargle could reduce BMS symptom temporarily [101, 102].

**Fig. 39:**
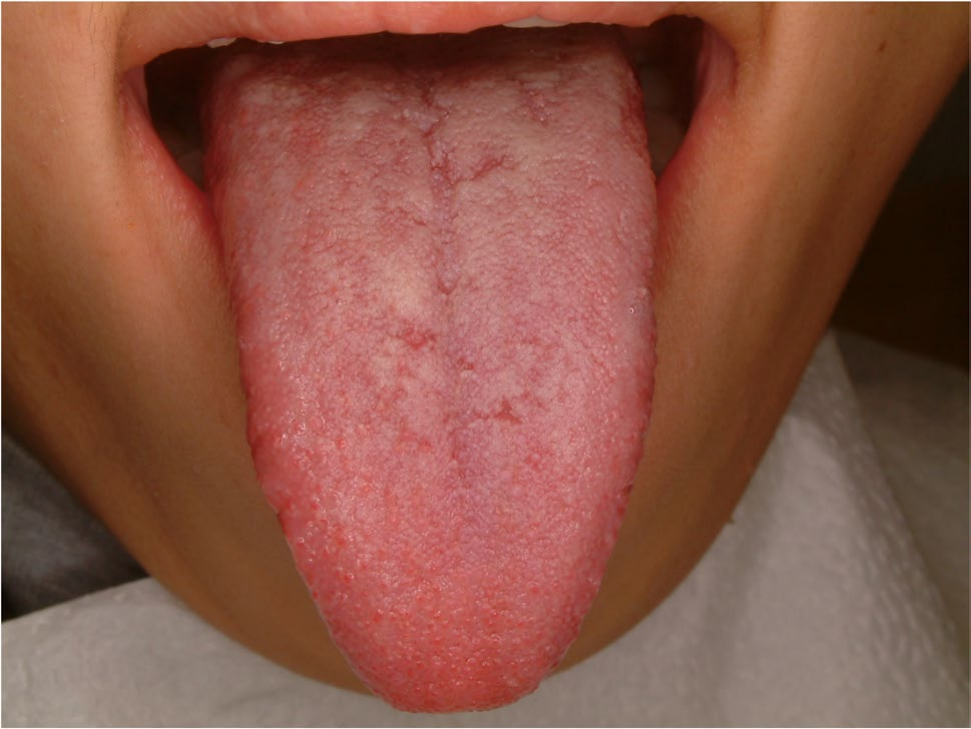
Tongue in burning mouth syndrome patient

#### Behçet’s disease (Fig. 40)

Behçet’s disease (BD) is one of common autoimmune disease which shows diverse clinical signs and symptoms [103]. BD is originally a systemic vasculitis disorder characterized by oral, ocular and genital ulcer and skin lesions, however, BD affects other organs such as vascular, gastrointestinal and neurological systems [104]. One of the well-known risk factor is HLA-B51 and ERAP1-Hap10 (endoplasmic reticulum aminopeptidase 1- haplotype 10) which recessively confers the highest risk for BD through CD8 T cell [105, 106]. Systemic medical treatment is recommended in severe case with corticosteroids, azathioprine, cyclophosphamides, cyclosporine A, interferon-alpha, and anti-tumor necrosis factor alpha agents [107]. Loss of vision and neurological disease are major causes of morbidity and disability in BD patient [104]. Oral ulcer could be improved by systemic steroid administration and local steroid gargle.

**Fig. 40:**
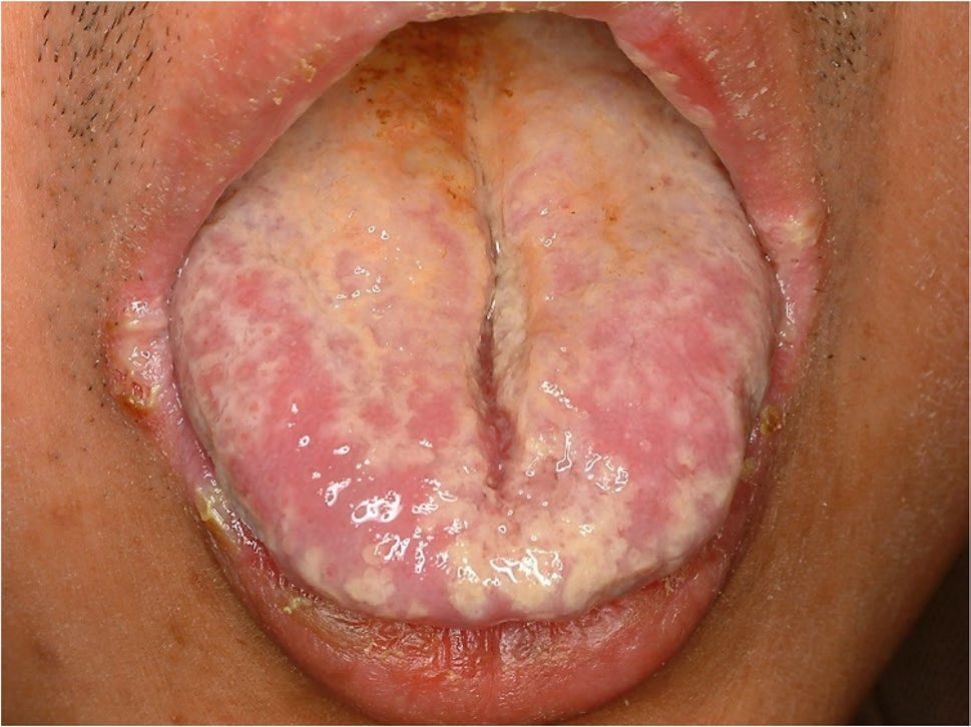
Necrotizing tongue mucosa in Behçet’s disease

## Conclusion

The tongue is a complex organ composed of muscles, mucosa and papilla. Diverse diseases can occur in the tongue, and accurate differential diagnosis is often challenging. This review classified 36 tongue diseases across infectious, inflammatory, neoplastic, precancerous, congenital, autoimmune, and systemic disease-related categories. Pathologic lesions generally require surgical removal or topical pharmacotherapy, whereas non-visible lesions presenting with discomfort and pain are typically associated with systemic or psychosocial conditions. Given the tongue’s critical role in speech, swallowing, and quality of life, individualized and multidisciplinary treatment planning is essential for optimal clinical outcomes.

## Data Availability

No datasets were generated or analysed during the current study.

## Abbreviations

MRG: Median rhomboid glossitis
EBV: Epstein-Barr virus
PA: Pleomorphic adenoma
SCC: Squamous cell carcinoma
KS: Kaposi sarcoma
CXPA: Carcinoma ex pleomorphic adenoma
ACC: Adenoid cystic carcinoma
UPS: Undifferentiated pleomorphic sarcoma
BWS: Beckwith-Wiedermann syndrome
IDA: Iron-deficiency anemia
TEN: Toxic epidermal necrolysis
BMS: Burning mouth syndrome
GABA: Gamma-aminobutyric acid
BD: Behçet’s disease
